# Unsupervised and supervised AI on molecular dynamics simulations reveals complex characteristics of HLA-A2-peptide immunogenicity

**DOI:** 10.1093/bib/bbad504

**Published:** 2024-01-16

**Authors:** Jeffrey K Weber, Joseph A Morrone, Seung-gu Kang, Leili Zhang, Lijun Lang, Diego Chowell, Chirag Krishna, Tien Huynh, Prerana Parthasarathy, Binquan Luan, Tyler J Alban, Wendy D Cornell, Timothy A Chan

**Affiliations:** IBM Thomas J. Watson Research Center, Yorktown Heights, NY 10598USA; IBM Thomas J. Watson Research Center, Yorktown Heights, NY 10598USA; IBM Thomas J. Watson Research Center, Yorktown Heights, NY 10598USA; IBM Thomas J. Watson Research Center, Yorktown Heights, NY 10598USA; IBM Thomas J. Watson Research Center, Yorktown Heights, NY 10598USA; Precision Immunology Institute, Icahn School of Medicine at Mount Sinai, New York, NY 10029; Broad Institute of MIT and Harvard, Cambridge, MA 02142, USA; IBM Thomas J. Watson Research Center, Yorktown Heights, NY 10598USA; Center for Immunotherapy and Precision Immuno-Oncology, Cleveland Clinic, Cleveland, OH 44195USA; Lerner Research Institute, Cleveland Clinic, Cleveland, OH 44015USA; IBM Thomas J. Watson Research Center, Yorktown Heights, NY 10598USA; Center for Immunotherapy and Precision Immuno-Oncology, Cleveland Clinic, Cleveland, OH 44195USA; Lerner Research Institute, Cleveland Clinic, Cleveland, OH 44015USA; IBM Thomas J. Watson Research Center, Yorktown Heights, NY 10598USA; Center for Immunotherapy and Precision Immuno-Oncology, Cleveland Clinic, Cleveland, OH 44195USA; Lerner Research Institute, Cleveland Clinic, Cleveland, OH 44015USA; Department of Radiation Oncology, Memorial Sloan Kettering Cancer Center, New York, NY 10065USA; Taussig Cancer Institute, Cleveland Clinic, Cleveland, OH 44015USA; National Center for Regenerative Medicine, Cleveland Clinic, Cleveland, OH 44015USA

**Keywords:** MHC–peptide complex, molecular dynamics, Markov models, graph convolutions, immunogenicity, cancer immunotherapy

## Abstract

Immunologic recognition of peptide antigens bound to class I major histocompatibility complex (MHC) molecules is essential to both novel immunotherapeutic development and human health at large. Current methods for predicting antigen peptide immunogenicity rely primarily on simple sequence representations, which allow for some understanding of immunogenic features but provide inadequate consideration of the full scale of molecular mechanisms tied to peptide recognition. We here characterize contributions that unsupervised and supervised artificial intelligence (AI) methods can make toward understanding and predicting MHC(HLA-A2)-peptide complex immunogenicity when applied to large ensembles of molecular dynamics simulations. We first show that an unsupervised AI method allows us to identify subtle features that drive immunogenicity differences between a cancer neoantigen and its wild-type peptide counterpart. Next, we demonstrate that a supervised AI method for class I MHC(HLA-A2)-peptide complex classification significantly outperforms a sequence model on small datasets corrected for trivial sequence correlations. Furthermore, we show that both unsupervised and supervised approaches reveal determinants of immunogenicity based on time-dependent molecular fluctuations and anchor position dynamics outside the MHC binding groove. We discuss implications of these structural and dynamic immunogenicity correlates for the induction of T cell responses and therapeutic T cell receptor design.

## INTRODUCTION

Immune surveillance is a continual process whereby short peptides, commonly 9–12 amino acids in length, are displayed on the cell surface via major histocompatibility complex (MHC) molecules. Proteasomal degradation of cytosolic proteins supplies the MHC molecules with a constant supply of peptides to load, with MHCs presenting those peptides that bind inside the MHC binding groove. Past studies have demonstrated that peptide binding to an MHC molecule, which is a prerequisite to T cell immunogenicity, is achieved through two buried peptide ‘anchor’ residues within the MHC peptide-binding pocket. Once a peptide forms a complex with an MHC, immunogenicity is thought to be determined by the identities of exposed peptide residues that form the primary epitope for T cell receptor (TCR) interactions [1, 2]. However, the processes that drive immunogenicity are not completely understood.

Immunogenicity prediction, along with understanding of the molecular mechanisms that drive such immunogenicity, has critical biomedical applications in vaccine and immunotherapy development. Artificial intelligence (AI) or machine learning models for MHC–peptide complex immunogenicity prediction have primarily focused on text-based amino acid sequence representations of isolated MHC-binding antigen peptides [3–5]. Over the years, these sequence-based models have improved our understanding of the anchor residue conditions that drive immunologic response to pathogen-derived peptides and have been used as an integral part of viral vaccine development. However, most of these models fail when they encounter smaller, real-world datasets or are asked to predict cancer-derived antigens. For example, in the Tumor Neoantigen Selection Alliance study, 28 separate groups were asked to predict cancer neoantigens and provide top therapeutic candidates. After screening these top candidates for true immunogenicity, only 6% of predictions were validated [6]. Peptide presentation to the adaptive immune system is a subtle molecular process with various structural and dynamical features appearing at the TCR-binding interface [7–10] that are not captured by sequenced based strategies. Initial efforts at integrating molecular structure and dynamics into immunogenicity prediction and interpretation have been limited in terms of AI methodology. Still, these attempts have revealed new correlates of immunogenicity that are difficult to access at the sequence level alone [11, 12].

Atomistic molecular dynamics (MD) simulations provide a rigorous approach for generating MHC–peptide complex conformational ensembles relevant to peptide presentation. In contrast to methods like rigid and flexible protein–peptide docking, MD trajectories allow MHC-bound antigen peptides full freedom to mold the MHC binding groove to match the specific antigen sequence and explore conformational presentation space in explicit solvent. The resulting equilibrated structural ensemble represents a peptide’s presentation landscape, containing the full range of sampled presentation modes and weights indicating which peptide arrangements are most likely to be exposed to TCR interactions. The inclusion of explicit solvent molecules is particularly important in the study of MHC–peptide complexes: hydrophobic exposure is thought to be a key driver of peptide immunogenicity, and the hydrophobic effect is notoriously difficult to capture without explicit solvent [13, 14]. While structure prediction methods like AlphaFold [15] promise to yield better estimates of native protein–peptide complex structures, such approaches still cannot rigorously capture diverse conformational ensembles and physical dynamics.

We here apply both unsupervised and supervised AI architectures to extract insights into MHC–peptide complex immunogenicity mechanisms based on MD trajectory data. Two classes of AI methods serve as the present focus: (i) unsupervised Markov models [16–19] that allow for the estimation of slow molecular kinetics and the generation of novel MD trajectories and (ii) supervised molecular graph convolutions [20–23] that allow for the integration of varying degrees of local molecular interactions into multiscale representations of structure. Markov models have been shown to capture processes that occur at orders-of-magnitude slower timescales than can be naively detected in underlying MD trajectories, and graph-based deep learning architectures can integrate phenomenological features like physical potential energy functions (resulting in so-called ‘potential nets’) to augment molecular classification applications and complement training on atomistic MD simulation data [20–22].

We show that our combined AI and simulation framework can highlight subtle but key determinants of peptide immunogenicity within the MD trajectory data and can, under conditions outlined below, provide significantly more predictive power over a baseline sequence architecture on peptide datasets. Markov models are first utilized to elucidate the role of conformational structure and dynamics in immunogenicity. Classification models are then built from large-scale MD datasets spanning thousands of peptidic antigens. Improved classification performance is observed under low-data conditions where the data are corrected for trivial sequence correlations. Making predictions on small training datasets is commonly the only option available for probing cancer neoantigens and less-studied HLA alleles. Our MD–AI results further highlight mechanisms of peptide immunogenicity connected to peptide anchor dynamics and peptide fluctuations at large. These insights highlight how MD can help predict and foster understanding of immunogenicity, and the methods developed here lay a framework for broad HLA allele studies to further elucidate mechanisms of immune responses and inform T cell therapies.

## RESULTS AND DISCUSSION

### Unsupervised AI for probing subtle characteristics of cancer neoantigen immunogenicity

Cancer neoantigens typically represent an opportunity for immunologic recognition of cancer cells via a peptide sequence that differs from a wild-type (WT) sequence at only one mutational locus. As such, differences in immunogenicity that drive cancer cell elimination are likely to be tied to subtle differences in molecular presentation induced by that single residual change. To probe the impacts of this type of mutation, we collected over 1 ms of MD data on a lung cancer neoantigen/WT peptide exemplar pair restricted to HLA-A2: FLIYLDVSV/FLTYLDVSV [24]. Individual MD trajectories were extended to 100–200 ns in length; to synthesize the massive trajectory ensembles needed to reach the millisecond timescale in aggregate, we constructed unsupervised AI models (Markov models) for both the neoantigen (MT) and WT systems using identical procedures based on the well-established tICA dimensionality-reduction method on protein and peptide dihedral angle dynamics.

### Analysis of slowest relaxation timescales observed in neoantigen and WT MHC–peptide complexes

**Figure 1:**
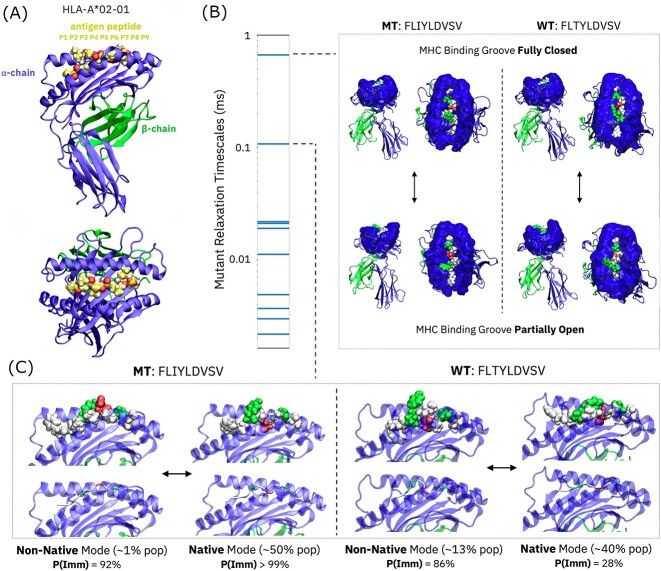
Slowest relaxation timescales estimated by unsupervised AI highlight concerted MHC–peptide dynamics and multiple peptide presentation modes. (**A**) Illustration of MHC–peptide complex system specific to the HLA-A2 supertype and representative of the cancer neoantigen/WT peptide pair under study. (**B**) MHC binding groove dynamics that represent the slowest timescale observed for both the neoantigen and WT peptides, with quantitative timescales presented for the neoantigen system. (**C**) Interconversion between peptide presentation modes that represents the second-slowest timescale observed for both neoantigen and WT peptides. *P*(Imm) values correspond to softmax outputs for each conformation derived from the best MD-graph immunogenicity prediction model presented in Section 2.

A typical presentation mode for an HLA-A2 peptidic antigen involves the burial or hydrophobic anchor residues at positions P2 and P9, with diverse residues potentially being presented at the peptide N-terminus (P1) and central positions (P3–P8) between the anchors (Figure 1A). Markov models allow for the estimation of exponential relaxation timescales that represent an entire trajectory ensemble rather than shorter, isolated trajectory components. The slowest processes observed in a model are often of biological interest, since slow conformational interchange can correspond with distinct conformational ensembles that can impact biological processes [16]. In these two cases of peptides bound to MHC(HLA-A2) molecules, the transition-matrix eigenvalues suggest similar values for the slowest two MT and WT timescales: the first around 1 ms and the second just above 100 μs (Figure 1B). Only MT timescales are shown in the figure, but each of the top two WT timescales (Figure S1) was estimated to be within 50% of its MT correspondent.

Transition-matrix eigenvector modes (both positive and negative) offer descriptions of the molecular processes underlying relaxation timescales. For both the MT and WT peptides, the slowest timescales relate to MHC-molecule dynamics: the peptide-binding groove of the MHC transitions from a ‘closed’ state in which the peptide N- and C-termini are largely obscured from solvent, and a partially ‘open’ state in which all peptide residues are more exposed to solvent. The peptide presentation modes in each of these ‘open’ and ‘closed’ states are approximately equivalent apart from modulations induced by conformational changes in the MHC α1 and α2 helices. These data indicate that Markov models can access not only access processes relevant to the presentation of bound peptidic antigens to T cell binding, but also to the loading and unloading of peptides onto/from MHC molecules themselves. Since peptide binding to MHC molecules is a prerequisite to immunogenicity, interrogation of loading/unloading mechanisms could contribute to a richer understanding of immune response induction.

The second-slowest timescales inferred for both MT and WT peptides correspond more directly to peptide presentation to T cells: each timescale represents an interconversion between distinct peptide presentation modes. In the case of the neoantigen (MT), a low-probability (~1% population) mode interconverts with the native presentation mode; in the WT, a near-native mode that resembles the dominant neoantigen presentation mode (in both backbone and sidechain configurations) interchanges with the native mode in which peptide residues are universally less exposed. Note that other presentation modes are of course present within the conformational landscape and represented by additional Markov states; these modes exchange with the native conformation in each case far more quickly than the modes presented in Figure 1C and are generally far closer to the native modes in peptide conformational space. For this reason (and others stated above), we elected to focus analysis on the slowest conformational interchanges between peptide presentation modes. Potential implications of these conformational changes for immunogenicity and therapeutic TCR design are discussed in a subsequent section.

### Mechanism of differential immunogenicity between neoantigen and WT peptides

**Figure 2:**
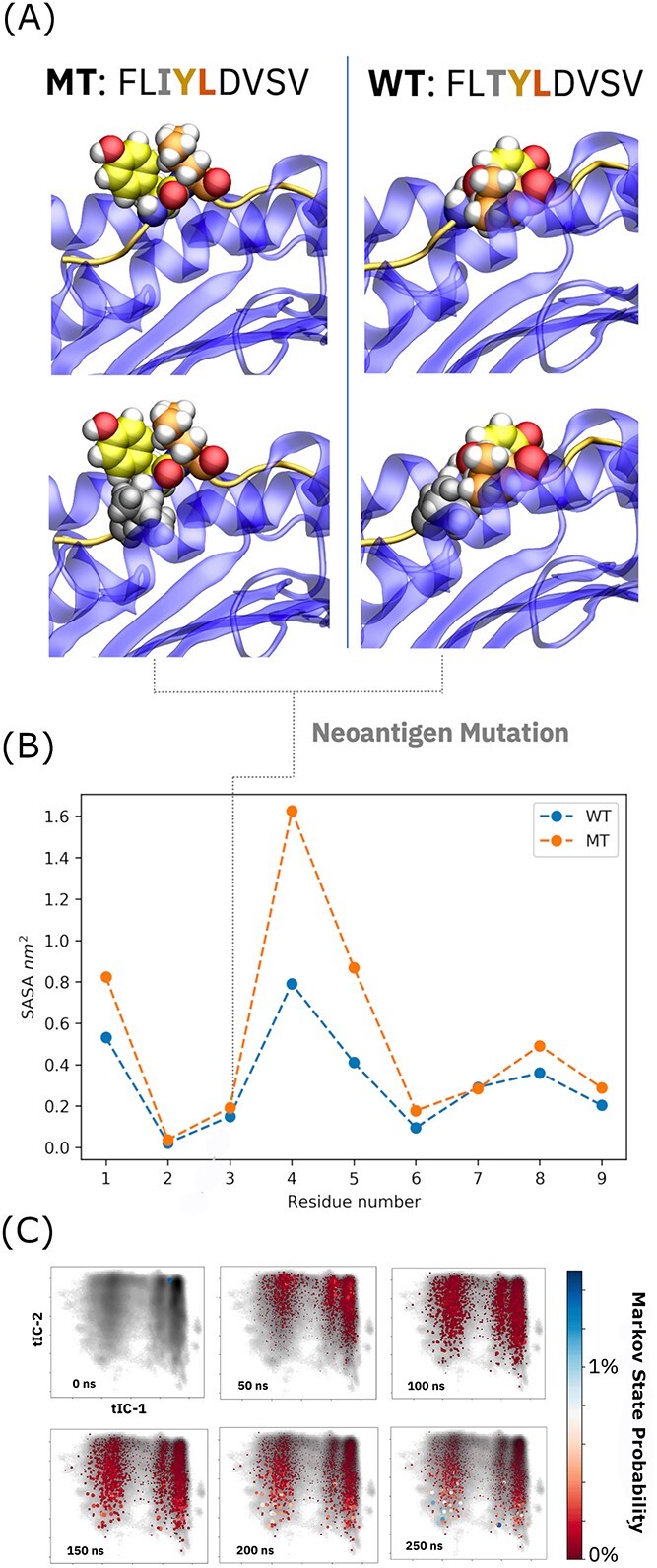
Presentation differences between cancer neoantigen and WT counterpart identified with unsupervised AI. (**A**) Dominant neoantigen and WT presentation modes drawn from the most populated Markov states of each model. (**B**) Quantitative estimates of residual solvent exposure differences between neoantigen and WT identified via population-weighted averages over the five most-populated Markov states in each model. (**C**) Equilibration of conformational populations propagated by each Markov model and projected onto top two time-components (tICs) obtained through dimensionality reduction of complex-wide dihedral angle vectors.

On average, the presented peptide conformations that dominate the MT and WT dynamical ensembles suggest a mechanism by which the FLIYLDVSV neoantigen is more immunogenic than its WT counterpart. Representative structures drawn from each Markov model’s most populous state (Figure 2A) are distinct. In the case of the neoantigen peptide, residues at P4 and P5 are thrust into solvent and made available for T cell interactions; in contrast, the predominant WT peptide is buried at P4 and P5, as facilitated by a more compact backbone configuration.

Past results have indicated that solvent exposure at P4 and P5, particularly in the context of hydrophobic/quasi-hydrophobic residues, is correlated with immunogenicity [8–12]. This surplus of solvent exposure at these key positions is likely responsible for differential neoantigen immunogenicity in this specific system and the efficacy of cancer immunotherapy in patients harboring this cancer mutation. Notably, the neoantigen and WT peptide only differ at P3 (threonine to isoleucine), a mutation that leads to virtually no conformational distinction at the point of change (Figure 2A). However, comparable burial of these beta-branched residues leads to dramatic changes in exposure for the middle of the peptide, reinforcing the hypothesis that subtle molecular phenomena can drive differential immune responses to the neoantigen in the FLIYLDVSV/FLTYLDVSV pair.

More quantitatively, an average of residual solvent exposure over the top five most-populous states in each Markov model shows that the mutated residues are almost identically buried, yet adjacent residues at P4 and P5 and significantly more exposed in the cancer neoantigen complex (Figure 2B). The Markov models provide unique access to these ensemble-wide observables: if one simply computes a conformational average over all frames collected in underlying trajectories of the MT complex (Figure 2C), the results converge to a basin that only reflects an unequilibrated starting configuration for peptide presentation. Over the course of ~250 ns of propagation using the AI model, the population density is diverted into the dominant, discrete states that describe the peptide-presentation landscape and peptide loading/unloading dynamics at large. Since it is not yet tractable to collect milliseconds of MD data on thousands of presented peptides, this local equilibration timescale of approximately 200 ns serves as a useful reference for simulations that can inform immunogenicity prediction based on molecular structure and dynamics.

### Potential implications of conformational immunogenicity for therapeutic TCR design

TCR design represents a key task in novel immunotherapeutic discovery that can benefit from understanding of TCR-peptide-MHC interfacial interactions [25] that are in turn influenced by presented peptide conformations. In the context of designing TCR molecules to recognize cancer neoantigens, one requires specific binding to the MT neoantigen peptide over its highly similar WT counterpart to achieve therapeutic efficacy.

As noted above, both the neoantigen and WT systems for FLIYLDVSV/FLTYLDVSV feature slow equilibration between presented peptide modes. Estimates of immunogenicity probabilities (Figure 1C) provide a preview of our second section focused on supervised immunogenicity prediction. As measured with our best-performing MD-graph immunogenicity prediction model trained on pathogen- and human-peptide data from the next section, the interconverting states in both neoantigen and WT complexes differ in terms of predicted immunogenicity. This observation represents a key point of emphasis in this work: peptides presented by MHC molecules aren’t innately immunogenic; rather, presented peptide conformations determine whether TCRs can bind.

In the case of the FLIYLDVSV neoantigen, the slow interconversion of presentation modes is predicted to occur between two highly immunogenic conformations. The FLTYLDVSV WT peptide, however, is predicted to present an immunogenic conformation remarkably like the neoantigen’s native presented state about 13% of the time. While the WT native state facilitates the sequestration of residues that are exposed in the neoantigen native state, the fact that a neoantigen-similar, likely immunogenic configuration appears in the WT presentation landscape with a large non-native population has serious implications for the negative selection of TCRs binding the FLIYLDVSV/FLTYLDVSV peptide pair. Slightly different MHC conformations connected to the MT/native and WT/non-native presentation modes might provide a narrow avenue for differential interactions. However, if one intended to design a therapeutic TCR to target the presented neoantigen, a receptor tailored to the native neoantigen presentation might select for the comparable WT configuration, as well. Targeting a non-native presentation mode thus might be necessary. In this case, a conformation with an ~1% population is likely targetable, given our prediction of ~100 μs kinetics for native/non-native mode interchange: a selective TCR should have ample time to encounter the non-native mode over the hours (or even days) of peptide presentation. These results highlight the need for understanding complex molecular mechanisms in the design of cancer immunotherapies. Similar analyses would need be conducted for arbitrary neoantigen/WT peptide pairs, as the current observations are not necessarily extensible to other systems; however, the current data provide a case study that could be applied to the full patient cohort presenting the FLIYLDVSV neoantigen.

### Supervised AI for peptidic-antigen immunogenicity prediction based on MD

Our results derived from unsupervised AI suggest that subtleties of molecular structure and dynamics can be critical to determining whether peptides are immunogenic, non-immunogenic, or sufficiently different in immunogenicity to effect a significant therapeutic response. While the above data facilitate a detailed interpretation of a system-specific, putative immunogenicity mechanism, supervised AI offers the benefit of scale for MHC–peptide immunogenicity predictions without the need for human intervention. We thus now detail our efforts to design an immunogenicity classifier that can take atomistic molecular features derived from MD simulations into account, using thousands of individual trajectories, about 200 ns in length, as a starting point.

### Analysis of massive MD trajectory ensemble

In seeking to use MD to model general MHC(HLA-A2)-peptide complex immunogenicity, we established a dataset of 2883 MHC–peptide complexes specific to the HLA-A02 supertype (containing pathogen/self-peptides curated from the IEDB [24, 26] and studied in previous work [12]; see SI for detailed descriptions of data and simulation characteristics). We chose to interrogate the HLA-A02 supertype because it matches our previous neoantigen case study and currently represents the best-characterized allele grouping with the most training data available for use. To facilitate a large-scale benchmarking of our MD classification methods, we collected ~200 ns MD simulations on all 2883 peptides. This choice of simulation length was informed by the local conformational relaxation profile probed via Markov modeling (Figure 2C), which suggests that the starting state tied to the template structure is largely forgotten at the 200 ns time point. We did also attempt to employ AlphaFold2-multimer to predict peptide presentation modes, but the output models failed to predict even the native presentation pose in template crystal structure (Figure S7). Others have recently reported similar difficulties in correctly predicting protein–protein complex structures tied to immunological systems with AlphaFold2-multimer [27].

**Figure 3:**
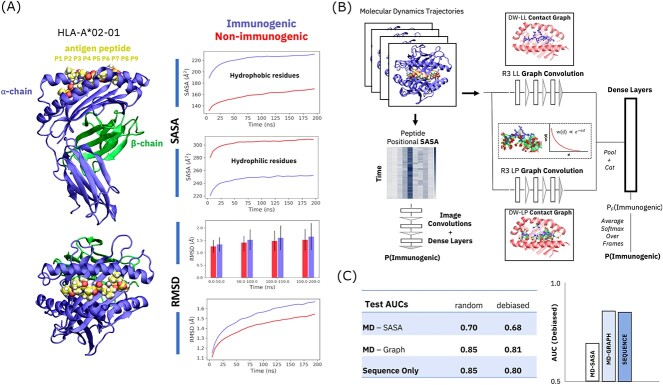
Classification of class I MHC–peptide complex immunogenicity based on molecular dynamics data. (**A**) MHC–peptide complex system subjected to MD simulation and class-differentiating observables distinguished by protein–peptide complex dynamics. (**B**) MD-graph deep learning architecture for MHC–peptide complex classification. (**C**) Performance comparison of MD-SASA, MD-Graph and sequence deep learning methods on the full A02 dataset.

An analysis of the nearly 1 ms MD trajectory ensemble for each complex highlights critical differences between the immunogenic and non-immunogenic peptide classes (Figure 3A). As observed in previous work, immunogenic peptides feature more hydrophobic surface area accessible to solvent. In contrast, nonimmunogenic peptides feature more exposure of hydrophilic surface area [8]. The surface exposure of hydrophobic residues is atypical of soluble protein exteriors and is a known biological signaling motif. On average, the peptide RMSD from each system’s respective starting state also tends to be significantly larger for immunogenic peptides. Both solvent-accessible surface area (SASA) and RMSD class gaps tend to widen as a function of simulation time, suggesting a possible advantage to training classifiers with MD data.

### Immunogenicity prediction with graph deep learning architecture

Molecular graphs encode the full complexity of molecular conformations and complexes, allowing deep learning architectures to benefit from features known to impact immunogenicity (like SASA as a function of peptide residue position) and distinct features that a practitioner might be unable to identify by default. Our graph-based deep learning architecture for MHC–peptide complex immunogenicity prediction features two molecular graphs: an intramolecular peptide contact graph (labeled LL, for ‘ligand–ligand’), and an intermolecular peptide-MHC graph (LP, for ‘ligand–protein’) (Figure 3B), that are input into separate graph convolutional encoders and combined to yield an immunogenicity prediction. For use as benchmarks, we adapt two immunogenicity classifiers presented in previous work: an MD-based architecture leveraging SASA features (Figure 3B) and a simple peptide sequence model. Our best-performing molecular graph architecture (hereafter: the MD-graph network) significantly outperforms the MD-only benchmark based on hand-engineered SASA features (Figure 3C). Across random dataset splits, the MD-graph network effectively equals the classification power of the sequence-only network on this full dataset. However, as detailed below, random dataset splits contain trivial correlations that can yield outsized performance in sequence-only architectures. Applying a Monte Carlo debiasing procedure that reduces sequence correlations between the largest training and test sets (see Methods), our MD-graph network modestly outperforms the sequence-only benchmark by 1 point. Predicted values for individual peptides come in the form of immunogenicity probability (softmax) values from both the sequence and MD-graph models and offer direct probabilistic estimates of model confidence in any one immunogenicity prediction. Distinctions in predictions on individual peptides can also distinguish the two approaches, as discussed below.

Benchmark AUCs for predictions on our 2883 peptide dataset generated with common, externally available immunogenicity prediction tools (NetMHCpan [28], MHCflurry [29], MixMHCpred [30], PRIME [31] and HLAthena [31]) are presented in Table S1. The five benchmark AUC values are all significantly below 0.5. This failure to classify our dataset perhaps relates to the fact that such external tools are often trained on MHC binding labels to predict binding as a surrogate for immunogenicity. Indeed, if one mixes peptides with confirmed negative A02 binding results into our dataset comprised of 2883 confirmed binders, the classification power of one example model, NetMHCpan, is restored to 0.72 (Table S1). One should thus potentially use caution in applying binding-prediction tools to immunogenicity prediction tasks when non-binding peptides are removed from the test set. These performance-related phenomena require further exploration in future work.

**Figure 4:**
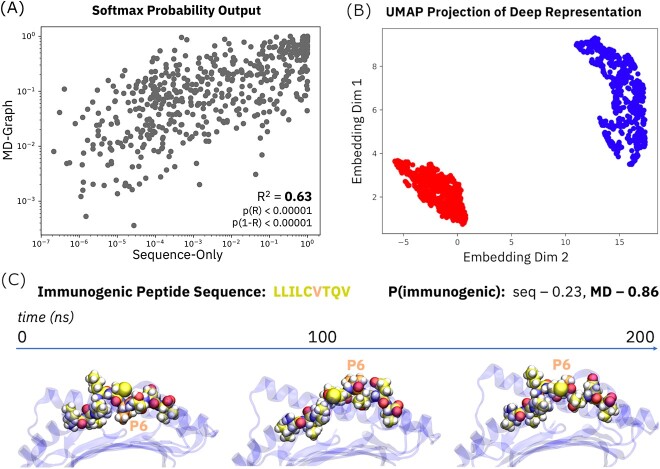
Comparison of predictions derived from sequence-only and MD-graph deep learning architectures. (**A**) Distinct predictions derived from MD-graph and sequence immunogenicity models, as represented by softmax values output from each model type. (**B**) Orthogonal UMAP projections of internal representation vectors derived from sequence and MD-graph model types. Sequence model projections are shown in red (lower left) and MD-graph model projections are shown in blue (upper right). (**C**) Anecdotal example of MD-graph rescue of a sequence model prediction in the context of peptide LLILCVTQV.

We observe that the immunogenicity predictions from our sequence and MD-graph models are only moderately correlated (*R*^2^ = 0.63; Figure 4A); a standard UMAP [32] dimensionality reduction across internal representation vectors (Figure 4B) illustrates similar orthogonality between MD-graph and sequence model predictions. These results suggest statistically significant variation exists between predictions from approaches that learn from MD and sequence features. Computed *P*-values for the Pearson correlation coefficient (*R*) and its symmetric complement (1-*R*) are both less than 0.00001, implying there’s little chance the two sets of predictions are completely uncorrelated or perfectly correlated. Figure 4C also illustrates a case in which the MD-graph model ‘rescues’ a positive prediction relative to the sequence model, wherein the MD-graph model yields a higher *P*(immunogenic) value for the immunogenic peptide LLILCVTQV. A dramatic conformational change typifies the presentation dynamics for that peptide: the valine at P6, which is initially buried, becomes exposed to solvent alongside the hydrophobic leucine at P4. This simultaneous presentation of hydrophobic residues at P4 and P6 is likely quite immunogenic, but the somewhat atypical nature of the presentation mode perhaps explains the sequence model prediction’s deficit.

These comparisons between MD-graph and sequence models motivate the creation of a combined MD + sequence model that combines the best of both feature sets to yield improved performance. Unfortunately, our efforts at this type of ‘multimodal fusion (MMF)’—which span simple concatenation schemes, framewise integration architectures and even cross-attention networks based on transformer models—have largely failed to improve on isolated MD-graph or sequence model results, to date. The SASA+sequence architecture described in previous work [12] also fails to improve on the isolated model results in this current test. We present our current best attempt that results from a framewise MMF architecture in Figure S8, an approach which yields a debiased test set AUC of 0.83 (representing a slight improvement over the respective sequence and MD-graph results of 0.80 and 0.81). Since this result sits on the border of statistical significance, we emphasize that future work is required to address this problem.

**Figure 5:**
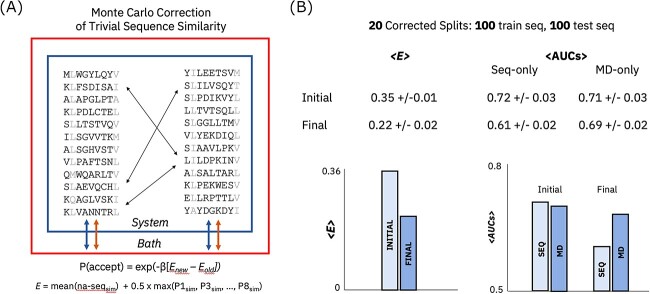
Superior MD model performance on smaller training data sets corrected for trivial sequence correlations. (**A**) Illustration of Monte Carlo approach to sequence split debiasing. (**B**) Sequence-only and MD-graph classification results comparison across 20 small datasets subjected to a correlation correction procedure.

Producing a massive training dataset is impractical for many applications (particularly cancer neoantigen classification [6]) given the cost and complexity of experimental data generation. We thus also apply a Monte Carlo split procedure (Figure 5A) to generate 20 pairs of training and test sets each containing 100 peptide sequences. Sequence similarity corrections on these small sets made sequence-only classification significantly more difficult: the mean AUC declined from 0.72 to 0.61 when compared with random data splits. Only a small decline is observed in MD-graph model performance (0.71–0.69), meaning the MD-graph model outperforms the present sequence model by eight points in AUC on the smaller debiased split ensemble (Figure 5B). This result provides encouraging evidence that MD-graph representations can be worthy of the added simulation cost in some application regimes. In practical scenarios where a test set overlaps heavily with a known training set distribution, sequence-only models are likely sufficient for immunogenicity prediction. A significant performance boost might be observed with MD-graph immunogenicity prediction models in more general situations lacking this overlap. In summary, sequence-only models should likely be prioritized in data-rich scenarios in which test examples have known similarity to training examples; our MD-graph could potentially benefit prediction efforts in low-data regimes with relatively novel test peptides. The MD-graph model also provides innate mechanistic interpretability, the implications of which being discussed below.

### Spatial and dynamical determinants of immunogenicity

**Figure 6:**
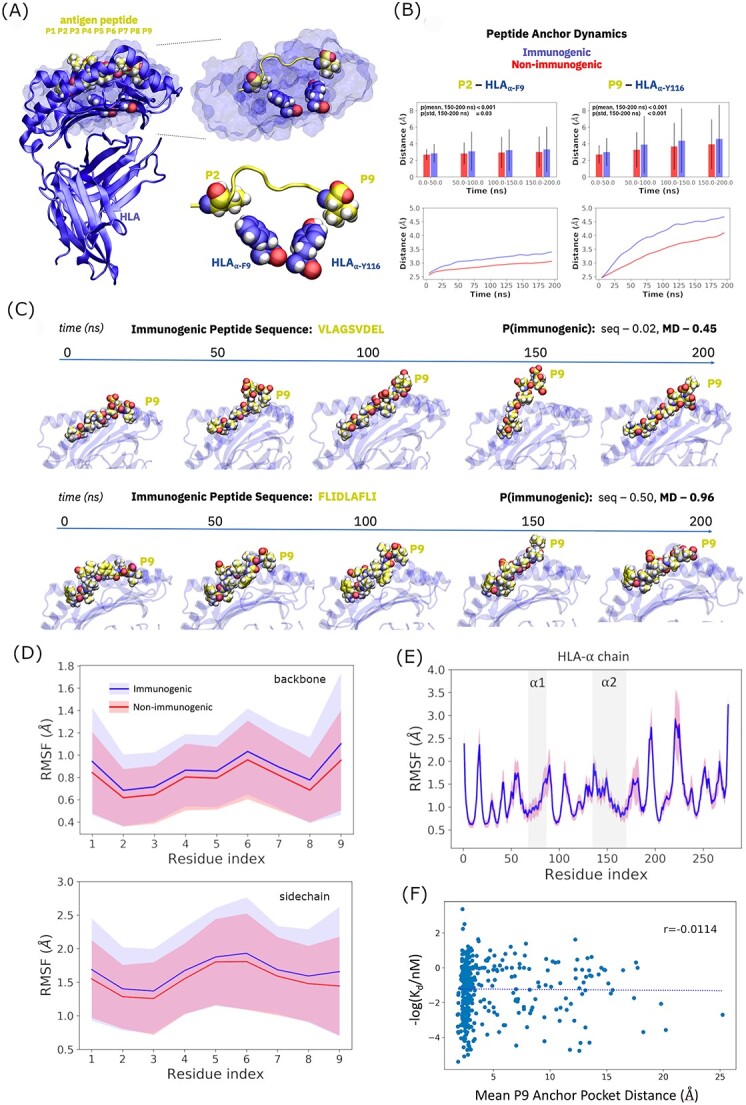
Anchor position spatial dynamics determine T cell immunogenicity. (**A**) Illustration of HLA-A02 supertype anchor binding pockets and HLA-A02-restricted peptide anchor positions. (**B**) Mean peptide anchor residue/MHC anchor pocket distances as a function of time, with error bars representing the full sampled conformational distributions. Computed *P*-values for mean peptide anchor distances and the anchor distance fluctuations (standard deviations; SDs) between the immunogenic and non-immunogenic peptide sets are presented in each subpanel for the 150–200 ns trajectory windows. (**C**) C-terminal peptide dynamics in two immunogenic peptide systems. Immunogenicity probability predictions are shown at right. (**D**) Backbone and sidechain RMSF values as a function of peptide position and immunogenicity class. Solid lines indicate mean values and shaded areas capture SDs. (**E**) Backbone RMSF values as a function of MHC-α position and immunogenicity class, with MHC groove helix residues highlighted with vertical shading and defined as being within approximately 8 Å of a reference peptide. Means and SDs are virtually indistinguishable between classes. (**F**) Correlation plot showing lack of relationship between quantitative binding affinity and P9 anchor dynamics.

Further analysis of ensemble-wide peptide RMSDs reveals that conformational deviations connected to immunogenicity can be attributed in part to the peptide anchor positions (Figure 6A). Minimum heavy atom distances between the P2 and P9 residues and their ⍺ chain interaction partners tend to increase, on average, over the course of simulation (Figure 6B), but much more so within the immunogenic peptide ensemble. The more dramatic difference appears at P9, where the mean distance gap between classes reaches approximately 1 Å across all 2883 MHC–peptide complexes. The differences in both mean anchor distances and the fluctuations in those distances are statistically significant at both P2 and P9 (with particularly high confidence at P9; Figure 6B). Accordingly, our data support the hypotheses that anchor residues of immunogenic peptides both spend significantly more time outside their binding pockets, on average, and tend to show larger fluctuations in their displacements from the HLA-A02 anchor pockets than non-immunogenic peptides.

These results indicate, in general, that the anchor positions are more dynamic and flexible in immunogenic peptides, particularly at the peptide C terminus. Instances in which the MD-graph model better predicts immunogenicity often correlate with striking dynamical events at P9 (Figure 6C): the peptide anchor at P9 dissociates from its MHC binding pocket, exposing much of the peptide C terminus to solvent (and potential TCR interactions) before reassociating. The MD-graph model thus appears to capture these anchor dynamics in its predictions, learning a complex dynamical determinant of immunogenicity from general intermolecular contact maps.

An analysis of peptide residue fluctuations (Figure 6D) shows that while the greatest dynamical gap between immunogenic and non-immunogenic peptides is observed at P9, immunogenic peptides feature more dynamic binding modes at every position. The backbone RMSFs at P9 are particularly large, highlighting the pronounced role of anchor dissociation in the overall dynamics. Fluctuations among tMHC binding groove residues (Figure 6E) do not markedly change as a function of peptide immunogenicity; it is noteworthy, however, that some MHC binding groove helix residues feature relatively dramatic fluctuations themselves, on average. To test the possibility that peptides with more dramatic anchor dynamics are simply weaker binders to the MHC molecule, we plotted our P9 anchor dynamics measure against quantitative MHC–peptide binding affinity measurements for the subset of peptides on which such quantitative values were available in the IEDB. As the plot (Figure 6F) shows, no such relationship can be observed, suggesting our MD simulations (and AI models designed to ingest them) are capturing subtle dynamical effects tied to immunogenicity.

Our results thus demonstrate clear advantages to using MD approaches for immunogenicity modeling when only small training datasets are available and when a greater insight into recognition mechanisms is desired, particularly in the context of peptide structure and dynamics. Unsupervised models have helped us reveal subtle determinants of neoantigen immunogenicity that should be likely considered in designing therapeutic TCRs against cancer peptides. And, in the context of supervised methods, could transient/partial unbinding events at peptide termini point to a mechanism for increased immunogenicity and TCR recruitment?

This study provides evidence to support both hypotheses, but establishing underlying mechanisms requires experimental confirmation. For example, all peptides within the supervised dataset are classified as MHC-binders; but once a minimum threshold for binding is reached, are more flexible and dynamic anchors naturally more immunogenic and attractive of TCRs? Additionally, conformational propensities for immunogenicity in the HLA-A2 supertype are likely not representative of all class I HLA alleles. Experimental tests of these hypothesized structural and dynamic determinants of immunogenicity need to be devised, but our current observations favor shifting antigen-selection procedures toward MHC–peptide complexes with properties like more flexible peptide termini and non-native presentation modes that can drive differential immune responses.

## METHODS

### MD simulation of MHC–peptide complexes

To facilitate unsupervised AI modeling, a neoantigen/WT peptide pair restricted to HLA-A2 binding (FLIYLDVSV/FLTYLDVSV) was simulated using the MD data collection procedure described below. The neoantigen complex was simulated with 3000 parallel trajectories, with each trajectory reaching 200 ns in length; as model construction determined approximately one-third of these MT data led to comparable results, only 1000 parallel simulations were conducted for the WT peptide counterpart.

For supervised AI modeling, HLA-A2 restricted peptides, numbering 2883 in total, were modeled into the HLA-A*02-01 peptide-binding groove based on a crystal structure template (PDB: 5NMH [33]) and using the VMD [34] Mutator plugin. The systems considered in this work were limited to peptides nine residues in length and correspond to a mixture of immunogenic peptides (*N* = 1038) drawn from pathogen sources and non-immunogenic HLA-binding peptides (*N* = 1845) drawn from human sources, as described in previous work [12]. MHC–peptide complex starting structures were then solvated in rectangular TIP3P water boxes with minimum extents of 12 Å from the nearest protein atom. Ionization with Na^+^ and Cl^−^ ions was carried out to neutralize the system and was extended to yield ionic concentrations of 150 mM. Using the NAMD [35] MD engine, the solvated and ionized systems were then subjected to steepest descent minimization for 10 000 steps and equilibrated at constant temperature (310 K) and pressure (1 ATM) for 20 ps with harmonic heavy atom constraints with a scaling factor of 25 kcal mol^−1^ nm^−2^ using the CHARMM36m [36] force field. Production runs were carried out for at least 200 ns. Temperature was controlled with a Langevin thermostat with a damping rate of 1/ps; pressure coupling was controlled with a Langevin piston barostat with a piston period of 100 fs and decay of 50 fs. Direct space interactions were computed with a 12 Å cutoff, and electrostatic interactions were handled using the Particle Mesh Ewald method [37] with a 1 Å grid spacing. Analysis of MD trajectory ensembles was computed with available tools in MDTraj [38] and VMD [34] libraries, with SASA values computed over full residues and RMSD values calculated across heavy atoms. Dihedral angle distributions were generated for the phi and psi angles of the peptide backbone.

### Markov modeling of MHC–peptide complexes

Markov models were constructed using the MSMBuilder 3.0 package [19] with default settings ascribed to tICA dimensionality reduction [17]. In brief, all applicable neoantigen and WT peptides were featurized to reflect protein backbone dihedral angles for each frame, as a function of time; these dihedral angle features were then subjected to the standard tICA dimensionality reduction procedure set to five-timescale parameterization. These tICA-based feature vectors were then clustered into 3000 discrete states using a simple *k*-means clustering algorithm, a procedure which minimizes the mean distances between individual tICA vectors and their nearest cluster centers. The *k*-means algorithm has been shown to be effective for clustering protein conformations after dimensionality reduction [17]. While other clustering algorithms (e.g. hierarchical, *k*-medoids) were also considered, the results of *k*-means yielded a favorable cluster center distribution that led to its selection.

Transition probability matrices for Markov models were created on this discrete state space using a reversible maximum-likelihood estimator and a common 25 ns lag time chosen via approximate convergence of implied timescale plots as a function of lag time. Transition matrix relaxation timescales were computed with standard linear algebraic transformations of matrix eigenvalues, and corresponding eigenvectors were projected onto tICs with direct state probability mappings.

### Deep learning architectures for immunogenicity prediction

Our molecular graph deep learning architecture was adapted from previous work and based on graph convolutions of local molecular structure [20, 21]. Two distance-resolved molecular graphs were constructed for encoder input, each with a global 8 Å cutoff: (i) an intramolecular peptide contact graph, or ligand–ligand (LL) graph, defined by contacts between heavy atoms within the peptide and not within two covalent bonded radii of one another; and (ii) an intermolecular MHC–peptide contact graph, or ligand–protein (LP) graph, including all contacts between all applicable MHC and peptide heavy atoms within the global cutoff. Graphs containing peptide covalent bonds were not included in our graph networks, since such information is trivially encoded in peptide sequence representations. Exponential distance weighting of these molecular graphs was implemented in a more sophisticated manner than in previous work [21] and further detailed in the Supplementary Information. Each distance-weighted graph was encoded through a series of three convolutional modules. Each convolutional module contained distance-bin submodules and was comprised of a neighbor atom convolution layer and corresponding neighbor atom pooling step. The outputs of the third and final convolutional modules (representing coarse-grained neighborhoods of three contact radii) were fed through a fully connected layer and subjected to gather operations across peptide atoms. The tensors returned by the gather operations were next pooled with maximum and average pooling operations, yielding an output embedding for each graph. These molecular graph embeddings were concatenated and fed through an additional dense (fully connected) layer prior to coupling with a binary softmax layer that produced an immunogenicity probability output.

Network training proceeded in TensorFlow [39] using a standard cross-entropy loss function over immunogenicity labels and an Adam [40] optimizer. Training set batches of 100 peptides were fed through the optimizer until the training loss converged, typically after approximately 25 epochs for the full training set and 140 epochs for the 100 peptide training sets. Peptide test sets were fed through the trained network in inference mode to yield softmax probability outputs, which were processed to estimate classification AUROC performance. The first 30 ns of each MD trajectory were excluded from training to accommodate an equilibration window.

Global hyperparameter optimization was carried out within a 2308 peptide training set generated through an initial application of the Monte Carlo debiasing procedure described in the Supplementary Information.

The reference MD architecture based on SASA features is described in the Supplementary Information. Our reference sequence classification architecture was also adapted from previous work [12]. Once more, in brief, peptide sequences were first encoded into one-hot representations ordered according to six standard residue similarity classes. These one-hot representations were then input into 2D convolutional layers covering both chemical (similarity) and physical (residue index) dimensions. Convolutional outputs were pooled and fed into two dense layers prior to softmax probability estimation. The training was also conducted in TensorFlow in parallel to the method described above, except with the replacement of the ADAM optimizer with stochastic gradient descent (which yielded more stable results).

### Monte Carlo correction for trivial sequence similarity

Split corrections based on Monte Carlo simulations were generated via a cost function, or energy function, *E*:


(1) \begin{equation*} E=\mathrm{mean}\left(\mathrm{na}-{\mathrm{seq}}_{\mathrm{sim}}\right)+{w}_{\mathrm{pos}}\ast \max \left(P{1}_{\mathrm{sim}},P{3}_{\mathrm{sim}},\dots, P{9}_{\mathrm{sim}}\right) \end{equation*}

with the first term accounting for the mean similarity between sequences at non-anchor positions and the second term accounting for the maximum sequence similarity at any one peptide position. The second term is needed because even in cases in which mean sequence similarity is minimized, similarity can remain high at a single peptide position and lead to trivial classification results. The positional weight, *w*_pos_, determines the relative importance of each energy term; a value of 0.5 was generally used in this work.

Monte Carlo simulations for correcting trivial sequence correlations are run with trial sequence exchanges between training and test sets, with acceptance dictated by a standard Metropolis criterion:


(2) \begin{equation*} P\left(\mathrm{accept}\right)=\exp \left(-\beta \left[{E}_{\mathrm{new}}-{E}_{\mathrm{old}}\right]\right). \end{equation*}

The exponential parameter β represents at artificial temperature that allows for sampling of diverse sequence sets rather than steepest-descent minimization of sequence similarities. Additional details are provided in the Supplementary Information.

**Key Points**

- Factors that determine T cell responses to pathogen and cancer antigens extend beyond the sequences of those antigen peptides and depend on the structure and dynamics of MHC-presented peptide modes.
- An unsupervised AI method (Markov modeling) can detect MHC–peptide loading and unloading dynamics that serve as the pre-requisite to immune responses and identifies non-native peptide presentation modes that could impact TCR cross-reactivity and therapeutic TCR design.
- A supervised AI method (classification with molecular graph convolutions) facilitates immunogenicity prediction based on structural and dynamical peptide features and highlights determinants of immunogenicity like unconventional conformational rearrangements and terminal excursions outside the MHC binding groove.

## Supplementary Material

MD_AI_BIB_SI_clean_R1_bbad504

Supplementary_File_all_sequences_labels_bbad504

## Data Availability

The full sequence and label set used in this work are provided in the Supplementary Information. MD trajectory data are available upon request.

## CODE AVAILABILITY

Code for featurizing molecular dynamics trajectories and running a trained MD-graph HLA-peptide complex immunogenicity prediction model in inference mode is available at https://github.com/BiomedSciAI within the immunogenicity prediction subrepository.

## Notes

^†^Biographical note: The authors span an interface between computational and biological research and represent the institutions of IBM Research, Cleveland Clinic, Icahn School of Medicine at Mount Sinai, Memorial Sloan Kettering Cancer Center and the Broad Institute of MIT and Harvard.
