# Supplementary material for: Unsupervised and supervised AI on molecular dynamics simulations reveals complex characteristics of HLA-A2-peptide immunogenicity
Source: Brief Bioinform. 2024 Jan 16;25(1):bbad504. doi: 10.1093/bib/bbad504 (PMC10793977; doi:10.1093/bib/bbad504)
Supplement: MD_AI_BIB_SI_clean_R1_bbad504 [file md_ai_bib_si_clean_r1_bbad504.docx]

**Supporting Information**: Deep learning on molecular simulations reveals spatially specific and dynamical determinants of MHC-peptide complex immunogenicity

Jeffrey K. Weber*, et. al

*Corresponding Authors. Email: jkweber@us.ibm.com, [chant2@ccf.org](mailto:chant2@ccf.org)

**Supplementary Text**

*Extended methods: molecular dynamics simulation of HLA-peptide complexes*

HLA-A02 restricted peptides, numbering 2883 in total, were modeled into the HLA-A*02-01 peptide-binding groove based on a crystal structure template (PDB: 5NMH^29^) and using the VMD^30^ Mutator plugin. The systems considered in this work were limited to nine residues and length peptides and correspond to a mixture of immunogenic peptides (N=1038) drawn from pathogen sources and non-immunogenic HLA-binding peptides (N=1845) drawn from human sources. A more detailed description of dataset curation can be found in previous work^12^, and the full set of peptide sequences and assigned labels used in this work are included in the Supplementary Information.

HLA-peptide complex starting structures were then solvated in a rectangular TIP3P water box with a minimum extent of 12Å from the nearest protein atom. Ionization with Na^+^ and Cl^-^ ions was carried out to neutralize the system and was extended to yield ionic concentrations of 150 mM. Using the NAMD^31^ molecular dynamics engine, the solvated and ionized systems were then subjected to steepest descent minimization for 10000 steps and equilibrated at constant temperature (310 K) and pressure (1 ATM) for 20 ps with harmonic heavy atom constraints with a scaling factor of 25 kcal mol^-1^ nm^-2^. Simulations were then run at production with a 2 fs timestep at the same constant temperature and pressure using CHARMM27 and CHARMM36m^32^ force field parameters for 100 ns and 200 ns, respectively. CHARMM27 data were collected to conduct a simple force field comparison (see SI for more details) and were not used to produce results featured in the main text. Temperature was controlled with a Langevin thermostat with a damping rate of 1/ps; pressure coupling was controlled with a Langevin piston barostat with a piston period of 100 fs and decay of 50 fs. Direct space interactions were computed with a 12Å cutoff, and electrostatic interactions were handled using the Particle Mesh Ewald method^33^ with a 1Å grid spacing.

Molecular dynamics data were collected on a combination GPU-containing Cloud and HPC compute nodes, with an approximate average production rate of 25 ns per system per today across the ~3000 HLA-peptide complexes and two molecular dynamics force fields. Final trajectory lengths ranged from 100 ns to approximately 180 ns for CHARMM27 (341 µs in aggregate) and 200 ns to approximately 340 ns for CHARMM36 (628 µs in aggregate). Due to compute system restrictions, simulations generally had to be restarted every 24 hours, meaning 4-10 restart iterations were typically needed to complete each production run. Jobs were executed using 4 CPU cores and 1 NVIDIA V100 or A100 GPU as a standard. Simulations conducted in the IBM Cloud were executed with a containerized version of NAMD; containerized jobs were distributed across available GPU-containing Cloud nodes and run until termination was enforced. Cloud jobs could frequently run for longer than 24 hours.

Analysis of molecular dynamics trajectory ensembles was computed with available tools in MDTraj^34^ and VMD^30^ libraries, with SASA values computed over full residues and RMSD values calculated across heavy atoms. Dihedral angle distributions were generated for the phi and psi angles of the peptide backbone.

*Extended methods: deep learning architectures for immunogenicity prediction*

Our molecular graph deep learning architecture was adapted from previous work and based on graph convolutions of local molecular structure^20,21^. Two distance-resolved molecular graphs were constructed for encoder input, each with a global 8Å cutoff: 1) an intramolecular peptide contact graph, or LL (ligand-ligand) graph, defined by contacts between heavy atoms within the peptide not within two covalent bonded radii of one another; and 2) an intermolecular HLA-peptide contact graph, or LP (ligand-protein) graph, including all contacts between all applicable heavy atoms within the global cutoff. Graphs containing peptide covalent bonds were not included in our graph networks, since such information is trivially encoded in peptide sequence representations.

Exponential distance weighting of these molecular graphs was implemented in a more sophisticated manner than in previous work^21^, leveraging concepts similar to those seen in other potential nets. Contacts from each of the two molecular graphs were binned in half-Å increments from the minimum contact distance to the global cutoff, as described by the distance bin vector, *d*:

*d* = [2.5 Å, 3.0 Å, 3.5 Å, 4.0 Å, 4.5 Å, 5.0 Å, 5.5 Å, 6.0 Å, 6.5 Å, 7.0 Å, 7.5 Å, 8.0 Å]

This distance vector was then subjected to a weighting operation defined by a single decaying exponential function, *w:*

$$w=\exp\left( -\alpha\cdot d \right)$$

where the decay rate ⍺ is chosen as part of the global hyperparameterization procedure described below. Optimal decay rates were found at values just under 0.5; a value of 0.41 was chosen for model training. Scalar weights output by this weighting function were then applied to the outputs of graph convolutional submodules corresponding to each distance bin, effectively downweighting the magnitudes of convolutional output as a function of increasing distance.

Each distance-weighted graph was encoded through a series of three convolutional modules. Each convolutional module contained distance-bin submodules and was comprised of a neighbor atom convolution layer and corresponding neighbor atom pooling step. The outputs of the third and final convolutional modules (representing coarse-grained neighborhoods of three contact radii) were fed through a fully connected layer and subjected to gather operations across peptide atoms. The tensors returned by the gather operations were next pooled with maximum and average pooling operations, yielding an output embedding for each graph. These molecular graph embeddings were concatenated and fed through an additional dense (fully-connected) layer prior to coupling with a binary softmax layer that produced an immunogenicity probability output.

Network training proceeded in TensorFlow^35^ using a standard cross-entropy loss function over immunogenicity labels and an Adam^36^ optimizer. Training set batches of 100 peptides were fed through the optimizer until the training loss converged, typically after approximately 25 epochs for the full training set and 140 epochs for the 100 peptide training sets. Peptide test sets were fed through the trained network in inference mode to yield softmax probability outputs, values which were processed to estimate classification AUROC performance. The first 30 ns of each MD trajectory were excluded from training to accommodate an equilibration window.

A detailed schematic of our molecular graph (MD-graph) architecture is included in Fig. S4. Performance improvements were observed after integrating exponential distance weighting and framewise ensemble averaging protocols (see SI for further discussion), implying that features only accessible through molecular structure and dynamics are augmenting the network’s classification power.

Global hyperparameter optimization was carried out within a 2308 peptide training set generated through an initial application of the Monte Carlo debiasing procedure described below. This training set was split into six subsets of equal size, and models were trained on all possible unique combinations of 5/6 subsets to perform a six-fold cross validation. Hundreds of variations of hyperparameters were sampled for the L2 regularization weight, distance decay rate ⍺, convolutional layer dimensions of the LL and LP graphs, and final (dense) layer dimensions. Sampling was conducted first on a discrete hyperparameter grid and in later iterations via a Gaussian process over each parameter of interest. Final network hyperparameters used in this work are presented in Fig. S4. A sequence model with a final layer dimension matching that of the MD-graph model was also trained to facilitate direct comparisons between MD-graph and sequence models via UMAP^25^; default UMAP parameters were applied across two embedding dimensions to create the plot shown in the main text.

Our reference architecture based on SASA features was adapted from previous work. In brief, MD trajectories were fed through a featurizer which computed the SASA of each residue as function of time; images containing these SASA values were then split into two channels based on hydrophobic (GAVLIFWYP) and complementary residue designations and fed through 2D image convolutional layers. Convolutional outputs were then pooled and input to two dense layers prior to softmax probability estimation. Training was conducted in TensorFlow in a parallel manner to that described above.

Our reference sequence classification architecture was also adapted from previous work. Once more, in brief, peptide sequences were first encoded into one-hot representations ordered according to six standard residue similarity classes. These one-hot representations were then input into 2D convolutional layers covering both chemical (similarity) and physical (residue index) dimensions. Convolutional outputs were pooled and fed into two dense layers prior to softmax probability estimation. Training was also conducted in TensorFlow in a parallel manner to that described above, except with the replacement of the ADAM optimizer with stochastic gradient descent (which yielded more stable results).

*Extended methods: Monte Carlo correction for trivial sequence similarity*

One mathematically rigorous approach to split debiasing involves the explicit generation of training and test dataset probability distributions with non-trivial overlap. Monte Carlo simulations, first conceived in statistical mechanics for sampling low free energy states in molecular systems, have emerged as a general framework for sampling probability distributions with flexible constraints. In the case of dataset splitting for HLA-peptide complex immunogenicity prediction, we wish to minimize trivial classification performance derived from simple sequence similarities within our dataset. We can apply this constraint through the definition of a cost function, or energy function, *E:*

$$E=\mathrm{mean}\left( na{\text{-}seq}_{sim} \right)+w_{pos}*\max\left( {P1}_{sim},{P3}_{sim},\ldots,{P9}_{sim} \right) (1)$$

with the first term accounting for the mean similarity between sequences at non-anchor positions and the second term accounting for the maximum sequence similarity at any one peptide position. The second term is needed because even in cases in which mean sequence similarity is minimized, similarity can remain high at a single peptide position and lead to trivial classification results. The positional weight, *w_pos_*_,_ determines the relative importance of each energy term; a value of 0.5 was generally used in this work.

Monte Carlo simulations for correcting trivial sequence correlations are run with trial sequence exchanges between training and test sets, with acceptance dictated by a standard Metropolis criterion:

$$P\left( \mathrm{accept} \right)=\exp\left( -\beta\left[ E_{new}-E_{old} \right] \right) (2)$$

The exponential parameter β represents at artificial temperature that allows for sampling of diverse sequence sets rather than steepest-descent minimization of sequence similarities.

This general Monte Carlo procedure was used on the full 2883 peptide dataset (seeded with a random 2308/575 peptide train/test split) to create the “debiased” training and test sets mentioned in the main text. Sequence exchange trials were conducted with 1-1 sequence swaps between training and test sets. The performance of both the sequence and MD-graph models declined on this set by approximately three points in AUC on this debiased set, as compared to the mean random result.

Monte Carlo simulations were carried out using the energy (cost) function and standard Metropolis acceptance criterion described in Eq. 1-2.

To efficiently compute the energy function for each sequence swap trial, matrices of mean peptide sequence similarities (calculated at non-anchor positions {1,3,4,5,6,7,8}) and matrices specific to each position were computed based on six standard residue similarity classes and imported. Random sets of peptides were then generated according to the desired training and test set sizes to seed the Monte Carlo sampling procedure. Peptide swap trials were carried out and evaluated based on the energy function, with the acceptance criterion determining whether 1) the swap was accepted and the simulation step incremented or 2) the swap was discarded and the step restarted. When additional peptides were available outside of the desired training and test sets, swaps were conducted both within the “system” (training/test sets) and between the system and “bath” (remaining sequences); when every peptide was included in either the training or test set, swaps could only occur within the system.

Artificial temperature values were set at levels that produced acceptance rates of 2-5% percent; typically, artificial temperatures of 0.25 supported this sampling regime. For both the full dataset correction and smaller (100 train, 100 test) dataset split generation, datasets were equilibrated in this moderate temperature regime for at least 2000 accepted steps, and additional steps were accepted for as long as reasonable (24 hour) simulation time allowed. The final configurations from this initial sampling step were then quenched for 100 accepted steps at a low temperature (0.05) to yield low energy configurations for use in model training. This sampling process was conducted 20 independent times to generate the small debiased split ensemble.

The procedure used to generate the full dataset correction was more complicated than that used for the smaller sets in one respect: to accommodate the 80%/20% train/test split but maintain equal-sized sequence sets within the code, the optimization was conducted across five peptide sets, four of which being merged to generate the final training dataset. An additional energy function weight (generally set to a value of 0.2) was applied to train/test cross-energy terms to break the degeneracy between the four sequence subsets merged for training and the fifth sequence subset designated as the test set.

*Additional background on T cell immunogenicity*

Adaptive immune responses mediated by T lymphocytes are critical to combat pathogens and neutralize tumor cells. T cell-dependent immunity is regulated by major histocompatibility complex (MHC)-presented peptides that are displayed on the surfaces of cells and subjected to surveillance. These MHC presented peptides are produced by proteasome-mediated digestion of proteins, which are subsequently loaded onto MHC complexes. Immunogenicity, defined by the extent of a stimulated T cell response, is determined, in part, by the residence time of T cell receptor (TCR) binding to HLA-peptide complexes, wherein slow TCR off rates are positively correlated with T cell activation. Interactions of the complex of MHC, peptide, and TCR form a core part of the “immune synapse” for distinguishing foreign antigens from self-antigens. MHC-I peptides resembling “non-self”– e.g., peptides derived from viruses or cancer cells – can stimulate cytotoxic killer lymphocyte (CTL) responses.

The majority of peptides presented by MHC-I are 9 amino acids in length, however peptides can vary in length between 8 and 12 amino acids. MHC-I peptide complex formation is dictated by the identity of the HLA-A, HLA-B, or HLA-C allele. In the setting of 9 residue long peptides and a typical allele, peptide binding is driven by two anchor positions, P2 and P9. HLA alleles have been granted “supertype” classifications based, in part, on subclasses of anchor residues that are compatible with their binding pockets. For example, the common HLA A02 supertype consists of HLA-A alleles that can generally accommodate hydrophobic amino acids at both anchor positions simultaneously. Peptide binding to an MHC complex is a prerequisite to MHC-dependent adaptive immunity. If a particular antigen peptide does not form a complex with an MHC, that peptide cannot be presented on the cell surface for interrogation by the immune system. Immunogenicity is determined primarily by the identities of the peptide residues that form the primary epitope for TCR interactions. Peptide presentation by the MHC drives the precise nature of interactions available to the TCR, with residues being exposed to solvent or buried within the MHC binding groove to partial or full extents as determined by non-linear correlations among non-anchor residues. Understanding the molecular basis of peptide-MHC:TCR interactions and developing approaches to accurately predict these interactions is one of the most important goals in immunology.

*Detailed perspective on immunogenicity prediction*

Immunogenicity prediction is of great interest in biomedical applications that involve immune-based mechanisms, like vaccines and immunotherapies. Peptide-MHC binding prediction models play an important role in identifying potential T cell epitopes. Sequence-based models for MHC-binding prediction have become multitudinous over the past three years, demonstrating usefulness for applications such as identification of immunogenic pathogen-derived peptides. Challenges such as decreased accuracy for less common HLA alleles and limited success for prediction of cancer neoantigens highlight the need for improvement. To date, models for immunogenicity prediction have largely focused on text-based, amino acid sequence representations of HLA-binding antigen peptides. However, this approach does not capture time-dependent effects. Peptide presentation on MHC complexes is a dynamic process with diverse structural and dynamical features appearing at the TCR-binding interface.

Initial efforts at integration of molecular structure and dynamics into immunogenicity prediction have revealed new correlates of immunogenicity that are difficult to access at the sequence level, such as hydrophobic residue exposure or HLA binding groove enclosure. While very important to the field, these first demonstrations have mostly been limited to small peptide sets, simple structural representations, and hand-engineered molecular featurization (like solvent-exposed surface area; SASA) procedures that rely heavily on human intuition. To advance these studies we propose the use of contemporary deep learning architectures designed for atomistic molecular structure and dynamics data focused on automated representation learning of atomistic coordinates. Since physical molecules exist in a dynamic three-dimensional space, string-based representations are undesirable for capturing many aspects of molecular interactions.

We here sought to utilize the molecular dynamics of MHC-peptide complexes to enhance our understanding of these fundamental interactions and train a model of immunogenicity that is superior to the standard sequence-based methods. In these efforts we complied a dataset of 3000 HLA-A02 MHC-peptide complex’s and built a distance-weighted graph convolutional neural network for the ingestion and classification of protein-peptide complex molecular dynamics data. We show that our molecular graph architecture outperforms models based on features derived from molecular dynamics data. Importantly, those our molecular dynamics model is independent from sequence-based models with increased performance. Together these data provide a framework for improving antigen prediction using molecular dynamics.

Networks designed for encoding molecular interactions, including sequence-to-structure prediction models like AlphaFold, have instead largely adopted combinations of image, graph, or distance map representations of molecules. While such spatial and spatiotemporal representations share commonalities, graphs are particularly natural molecular representations. Graphs and molecules share a structure of nodes as atoms and edges and bonds, and graphs have desired symmetry properties like translational and rotational invariance. Graph convolutions allow for the mixture of varying degrees of local molecular interactions into multiscale representations of structure; integration of physical potential energy functions into graph neural networks (resulting in so-called “potential nets”) has also been shown to be straightforward and effective in molecular classification applications.

While various simulation methods can be used to generate input structures, atomistic molecular dynamics simulations provide a rich but computationally expensive standard. Though approaches like protein-peptide complex structure prediction and protein-peptide docking can provide estimates of the native presentation modes of antigen peptides in HLA-peptide complexes, such conformational sampling is almost always more approximate than can be achieved with *ab initio* molecular dynamics force fields and integrators. Concepts like hydrophobic exposure of specific residue side chains to solvent, which could be critical for determining immunogenicity, are notoriously difficult to capture without explicit solvent dynamics. Furthermore, static structural methods generally cannot describe explicit protein dynamical effects like conformational exchange and partial unbinding that could very well impact the degree of immune response.

Although atomistic MD simulations are resource intensive, ever-improving high performance computing (HPC), cloud, and ASIC hardware resources are making MD simulations increasingly tractable at scale. Development of AI-surrogate models for molecular dynamics simulation (e.g., Markov models) promise further acceleration. Simple methods for encoding molecular dynamics trajectories into AI frameworks (e.g., time/ensemble averages) have deep roots in statistical mechanics, though more complex time encodings can be devised when desired.

*Supplementary figure for timescale estimation in unsupervised AI*

*
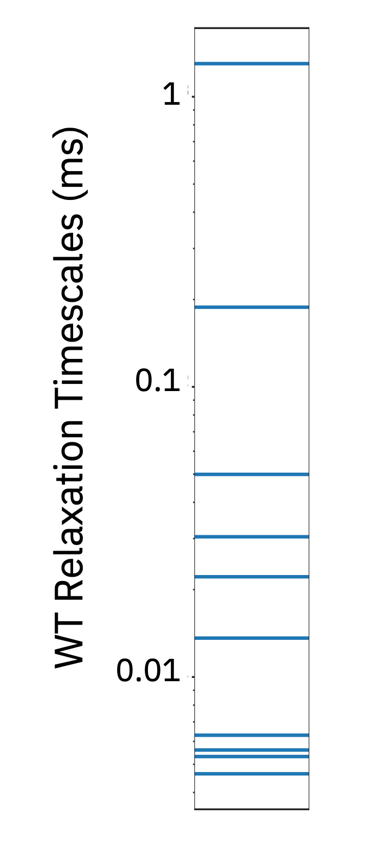
*

**Fig. S1. Slowest relaxation timescales for WT (FLTYLDVSV) MHC-peptide complex**. Timescales are estimated from transition matrix eigenvalues.

*Detailed analysis of molecular dynamics data for supervised AI*

To facilitate a large-scale benchmarking of our molecular dynamics classification methods, we collected molecular dynamics simulations on thousands of HLA-peptide complexes, with each of the nine-residue peptide systems restricted to the HLA-A02 supertype and labelled according to a positive or negative tetramer assay result as a measure of immunogenicity. This specific A02-restricted peptide dataset was curated in previous work from the IEDB and consists of 1038 immunogenic peptides drawn from pathogen sources and 1845 nonimmunogenic peptides drawn from human host sources, yielding 2883 peptides in total.

Each peptide system was loaded onto the specific HLA-A*02:01 allele (Fig. S1) using a crystal structure template, energy minimized, equilibrated with harmonic heavy atom restraints, and run at production until approximate local equilibration of peptide conformations was reached. Simulations were repeated in two molecular dynamics force fields – CHARMM27 and CHARMM36 – for at least 100 ns each to facilitate a detailed methodological comparison.

The two force fields yielded mutually consistent results with respect to the sampled space of peptide configurations, with a few substantial differences in dihedral angle space. Interestingly, no significant difference was observed in classification power between feature sets derived from each force field using the deep learning methods detailed later in the paper. This result suggests that both force fields’ conformational ensembles adequately captured features used to predict peptide immunogenicity, and that deep learning architectures may have a normalizing effect on molecular force field differences.

Given that CHARMM36 is the more modern force field, we focused our subsequent work on the CHARMM36 trajectory ensemble. Since both trajectory ensembles seemed at the borderline of local equilibrium based on standard order parameters like SASA and heavy atom coordinate root mean square deviation (RMSD), we elected to extend the CHARMM36 trajectories to at least 200 ns in length prior to further analysis. Global equilibration, which includes HLA conformational change and peptide unbinding/rebinding events, is expected to require far longer than 200 ns to reach; however, 200 ns seemed to provide a reasonably converged picture of peptide configurations within the local bound state. In aggregate, we thus collected nearly 6000 trajectories summing to nearly 1 ms in length (Fig. S1). To collect this volume of molecular dynamics data, we relied on a combination of HPC and Cloud compute resources, with distributed HPC and Cloud workflows subject to daily resource and time limits mimicking the more advanced orchestration technologies currently available through IBM’s Accelerated Discovery platform (Fig S1).

A simple analysis of CHARMM36 trajectories highlights key differences between the immunogenic and non-immunogenic peptide classes (Fig. S2). As observed in previous work, immunogenic peptides feature far more hydrophobic surface area accessible to solvent, whereas nonimmunogenic peptides feature far more exposure of the hydrophilic surface area. The surface exposure of hydrophobic residues is atypical of soluble protein exteriors and is a known biological signaling motif. In kinases, for example, phosphorylation often leads to the exposure of hydrophobic residues on a cytoplasmic protein tail that facilitates binding of signaling pathway partners. The peptide RMSD from each system’s respective starting state also tends to be significantly larger for immunogenic peptides, on average; conformational characteristics that underlie this difference in RMSD are detailed at the end of the Results and Discussion section. Both SASA and RMSD class gaps tend to widen as a function of simulation time, suggesting mechanisms through which molecular dynamics-derived features could offer a classification advantage over simple peptide sequence features or static HLA-peptide complex structural features.

*
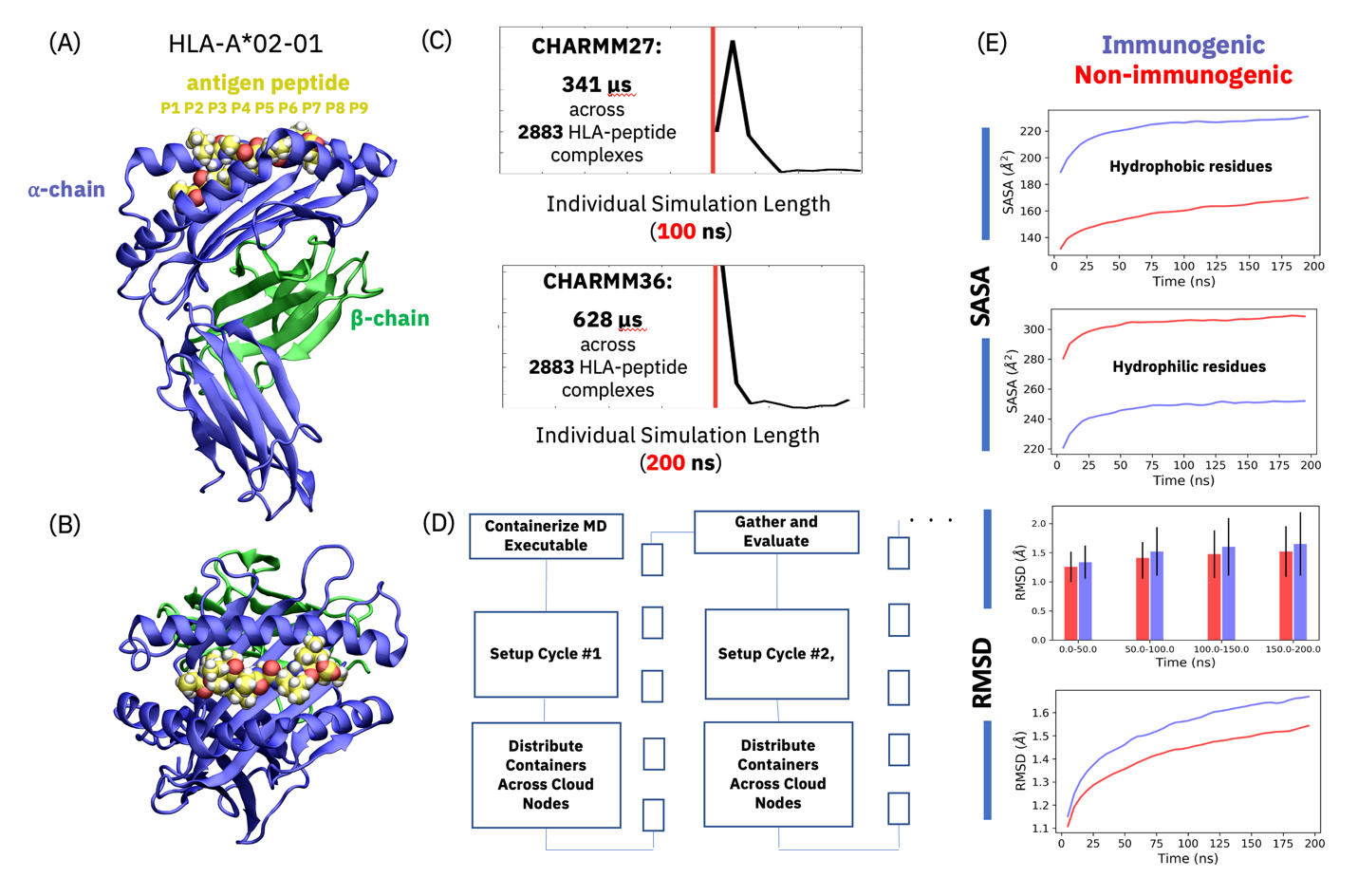
*

**Fig. S2 Exhaustive simulations of HLA-peptide complex ensemble**. Histograms of molecular dynamics trajectory lengths collected with two force fields, with vertical red lines indicating 100 ns or 200 ns time points; diagram of simple Cloud distribution workflow used to collect molecular dynamics simulation data. (E) Mean values of SASA and RMSD order parameters as function of time averaged over all HLA-peptide complexes belonging to each immunogenicity class.

Residue-specific differences in solvent exposure and conformational sampling are also readily accessible through analysis of the full CHARMM36 trajectory ensemble (Fig S2). In terms of solvent exposure (Fig. S2), residues at positions P4, P5, and P8 are most exposed in both immunogenic and nonimmunogenic peptides (Fig. S2), with notable exposure distribution differences between classes at each of these key peptide positions. Hydrophobic exposure trends at P4, P5, and P8 match the mean behavior seen in in the main text with near uniformity (Fig. S3). Hydrophobic exposure at these specific positions has been connected to enhanced immunogenicity in the literature. Residues at the first anchor position, P2, feature very little solvent exposure in either class, as expected; exposure at the adjacent P3 is also limited in both immunogenic and non-immunogenic peptides. Significant solvent exposure tails and class-specific exposure surpluses appear at P1, P6, and P7, suggesting some availability for differential TCR interactions at those positions. The solvent exposure distribution of the second anchor position, P9, also features a noticeable tail with an immunogenic peptide surplus, a fact that is revisited in more detailed analysis later in this section.

Distributions of backbone phi and psi dihedral angles indicate the conformational preferences of peptides within each immunogenicity class. Immunogenic and non-immunogenic peptides tend to populate similar conformational basins in some cases, with dense population in the extreme upper left quadrant at most positions that indicates a uniform up-down alternation of backbone carbonyl orientations. However, dihedral angles are only tightly localized in this quadrant at P2 and P3, with much more conformational variance at P4-P8. This result suggests a diverse conformational ensemble with respect to peptide presentation is accessed outside the extreme peptide N-terminus. Dihedral basin populations seem to deviate more from local maxima in non-immunogenic peptides at P4 and P5, implying that immunogenic peptides feature more stable and uniform presentation at these central peptide positions. The plots in Fig. S2-B show mean phi-psi values for each of the peptides in the 2883 peptide set. Dihedral variations within an individual peptide’s conformational ensemble that are uniquely derived from molecular dynamics could also be differentiators for immunogenicity prediction.

**
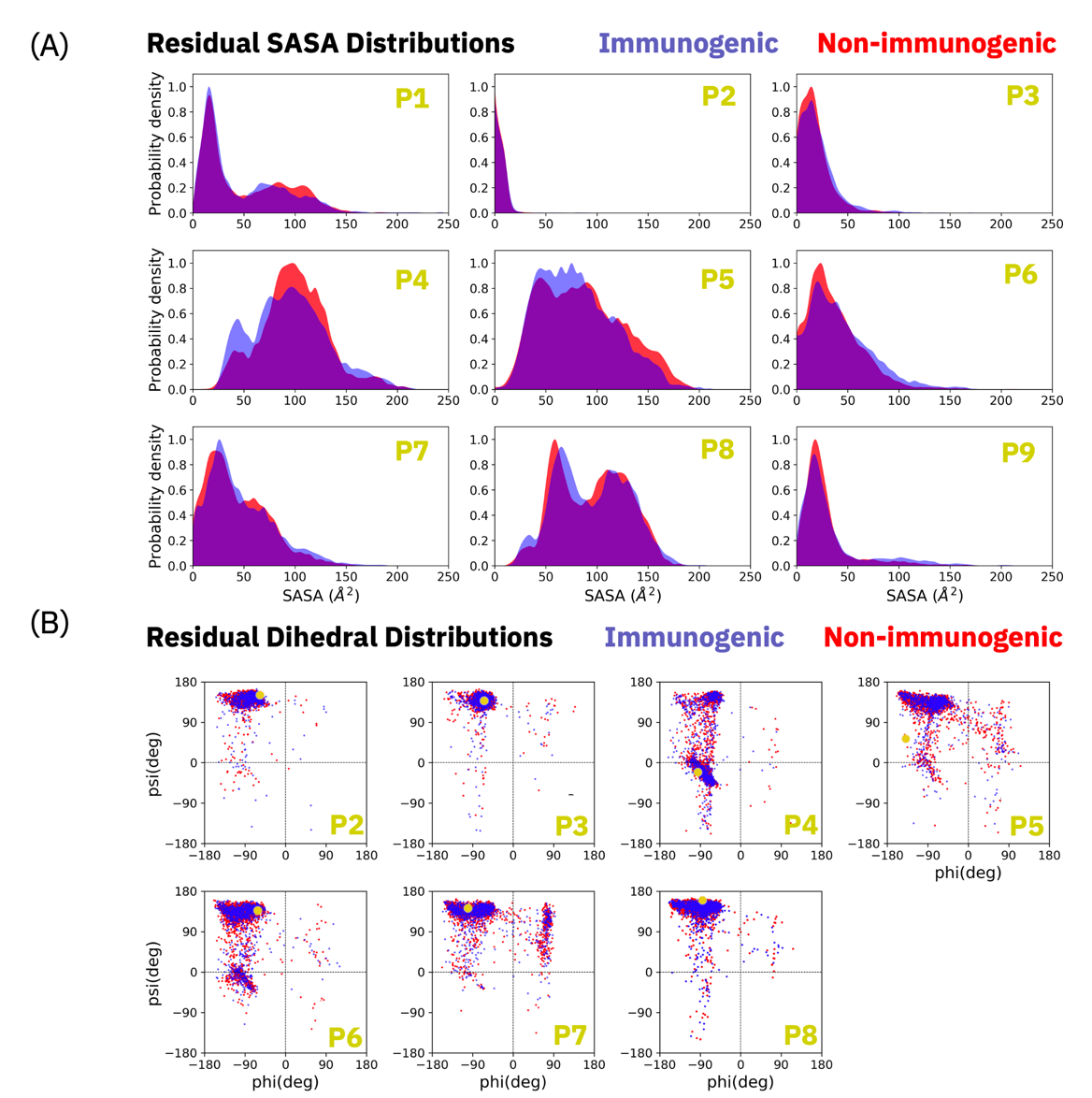
**

**Fig. S3** **Residue-specific characteristics of HLA-peptide complex ensemble dynamics**. (A) Solvent exposure distributions of immunogenic and non-immunogenic peptides at peptide positions P1 – P9. (B) Distributions of backbone phi and psi dihedral angles for the two peptide classes, with each dot colored by class and representing the mean for a single peptide. The initial dihedral angles for each peptide (corresponding to the angles observed in the PDB: 5NMH crystal template) are marked with gold dots.


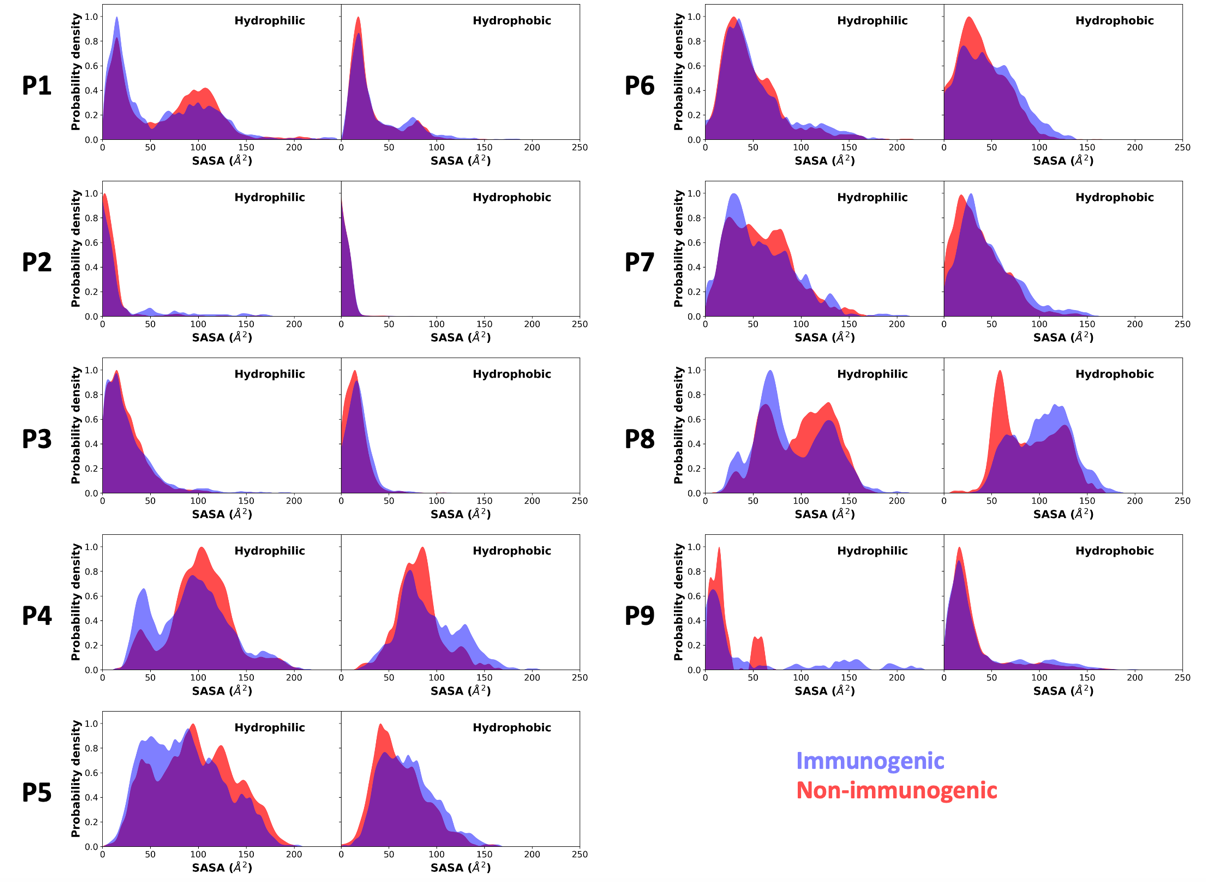


**Fig. S4. Residue solvent exposure divided among hydrophobic and hydrophilic and immunogenic and non-immunogenic classifications.**

*Further details surrounding immunogenicity prediction with distance-weighted gCNNs*

The above trajectory analysis suggests that MD trajectories can differentiate immunogenicity classes in unique ways. However, machine/deep learning classifiers are essential for automating immunogenicity prediction and rigorously demonstrating the value of MD feature sets. Classifiers that leverage MD data will here take the general form of many machine learning classifiers, containing: 1) a featurizer, which produces an initial representation of the data that can be read into the network; 2) an encoder, which generates a deep representation of those features, and 3) additional layers (or minimally, a single softmax layer), which produce a class probability output.

For use as benchmarks, we adapt two immunogenicity classifiers presented in previous work. The first, based on HLA-peptide complex MD data and inspired by observations of differential solvent exposure, relies on a positional SASA featurizer and a 2D space-time image convolutional encoder. The second, based on simple peptide sequence data, depends on a similarity-ordered one-hot vector featurizer and a 2D physico-chemical image convolutional encoder. The principal goal of this work is not to provide an exhaustive immunogenicity classification performance comparison across existing methods. The aim is rather to suggest where molecular graph-based architectures might offer distinct or complementary predictions from/to hand-engineered MD features and sequence-only architectures. Accordingly, the benchmarks presented here are derived only from the representative sequence and SASA networks described above.

The SASA-based MD classifier relies fully on human intuition in its construction: based on the hypothesis that differences in residue exposure are responsible for immunogenicity, the rich MD data are reduced to arrays of residual solvent exposure versus time. While this extreme hand-engineering of features can work well in specific classifiers, such drastic, intuition-based dimensionality and feature reduction contradicts the philosophy of representation learning that has come to dominate deep learning approaches. In representation learning, features are typically retained at a high level of complexity, with a sophisticated encoder performing a dimensionality reduction fixed only to the network’s loss function. This strategy allows for the deep representation of features that human intuition might overlook, often resulting in superior network performance.

Molecular graphs coupled to graph convolutional (or even more modern graph deep learning) encoders offer an attractive framework for representation learning on molecular dynamics data. Graphs of heavy atoms and their connections – either chemical bonds or intra/intermolecular contacts – provide input features at a highly general level, representing the full atomistic system with an internal coordinate system. The graph convolutional encoders featured in this work serve to mix atomic features up to a defined bond/contact radius, creating multiscale representations of the molecular system deep within the neural network.

In the context of HLA-peptide complex immunogenicity prediction, we construct two molecular graphs for input into a graph convolutional encoder: an intramolecular peptide contact graph (labeled LL, for “ligand-ligand”), and an intermolecular peptide-HLA graph (LP, for “ligand-protein”). The LL graph provides a detailed representation of the peptide’s conformation, while the LP graph captures both peptide interactions with the HLA and lack-thereof, in the context of residual solvent exposure. No graph including peptide chemical bonds is integrated into the network, as such information is trivially captured in peptide sequence features.

These molecular graphs are also distance-weighted in the spirit of potential nets. Contacts are placed in distance bins with half-Angstrom resolution up to a global 8Å cutoff, and scalar weights are applied to each distance bin with a decaying single exponential meant to mimic a physical potential energy function. The decay constant, ⍺, converged to approximately 0.5 in hyperparameter optimization; when plotted and scaled alongside attractive van der Waals and electrostatic potential terms typical of an MD force field, the single exponential curve falls in the middle.

The two molecular graphs are both fed through radius-3 graph convolutional modules, meaning three degrees of neighboring molecular contacts are mixed in the encoding process. The encoded representations of the LL and LP graphs are then concatenated, fed through dense neural network layers, and subjected to a binary softmax layer to produce immunogenicity probability predictions for individual MD frames. These framewise immunogenicity predictions are then ensemble-averaged to produce a single immunogenicity prediction per peptide system. Simplified component networks in which 1) exponential distance weighting is not applied, and 2) the ensemble average over framewise predictions is not conducted were also constructed for diagnostic purposes. The ‘no-distance weighting’ model is found to yield poor performance and large variations in AUC, likely arising from poor handling of conformational fluctuations due to the lack of distance modulation in the network. On the full dataset debiased split featured in Fig. 1c, the framewise average yielded an improvement of 3 points in AUC (0.78, F vs. 0.81, avg.). Ensemble averaging over frames provides a clear performance boost in AUC over performance on isolated frames. A sequence model with a final layer dimension matching that of the MD-graph model was also trained to facilitate direct comparisons between MD-graph and sequence models via UMAP.

The full 2883 HLA-peptide complex dataset was portioned into various 2308 peptide training and 575 peptide test sets, with splits carried out 1) with a simple 4-replicate random procedure and 2) a debiasing procedure intended to correct the split for outcomes based on trivial sequence similarity. This similarity correction procedure is presented in greater detail later. Hyperparameter optimization was carried out with six-fold cross-validation within the debiased peptide training set, and classification results are reported as simple AUC (area under the receiver operating curve, AUROC) values on respective held-out test sets. A detailed schematic of our network and hyperparameter choices are shown in Fig. S4.


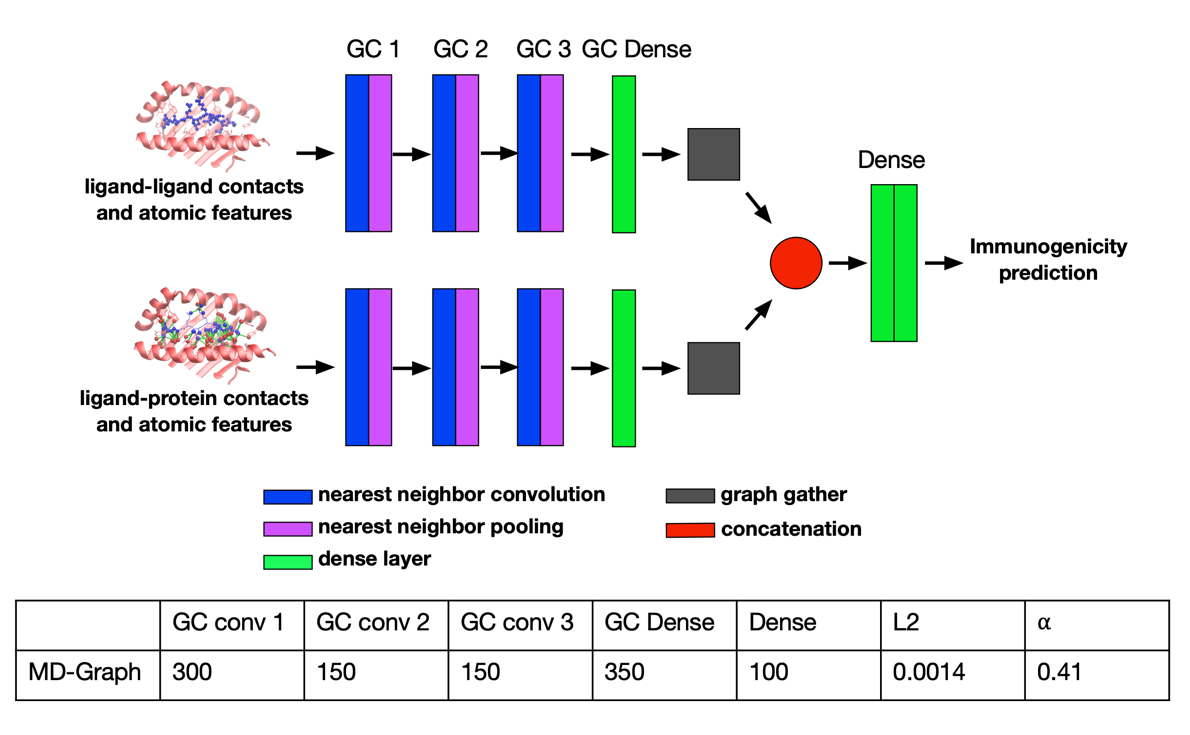


**Fig. S5. Detailed schematic of MD-graph network architecture and hyperparameter choices.**

*Learning curve for MD-graph method*

The MD-graph model learning curve indicates (Fig. S5-A) that classification power with the best MD-only model does not plateau until the order of 1000 training examples is reached. Model performance is generally unreliable when training sets contain a single-digit number of HLA-peptide complexes, reaching just above a mean AUC of 0.60 at n = 36. Test AUCs vary between 0.70 and 0.80 within the low- to high-hundreds of training examples. This regime might represent a favorable zone for MD-graph classification, considering the expense of generating experimental labels and the MD trajectories themselves on many more systems. Looking at distributions of test results based on dozens of random splits (Fig S5-B), model performance varies dramatically depending on the precise composition of the training set when single-/low double-digit training examples are used. The distributions begin to converge to more consistent results in the low hundreds of training examples.

**
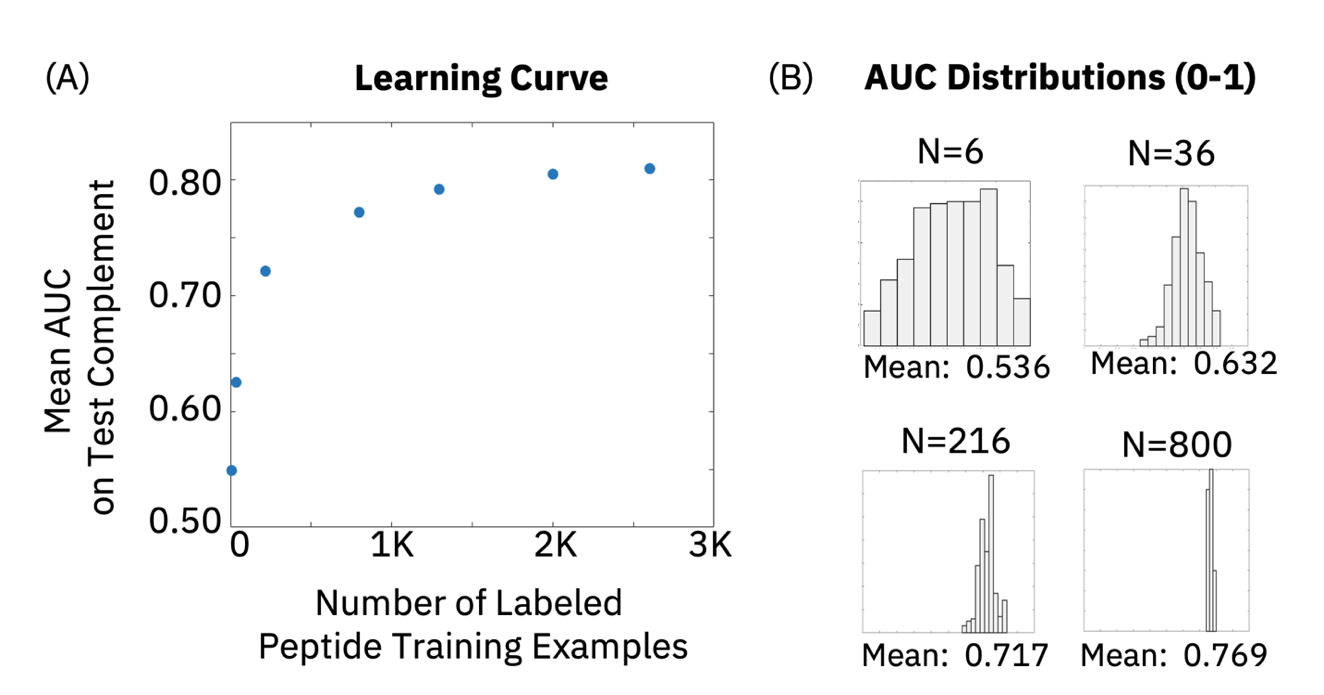
**

**Fig. S6 Learning curve analysis of MD-graph network performance**. (A) Learning curve points for the independent MD-graph model with training set sizes of [6, 36, 216, 800, 1296, 1600, 2000, and 2500] HLA-peptide complexes on a selected train/test split. Results were computed prior to hyperparameter optimization. (B) AUC distributions for dozens of random training sets corresponding to the first four points of the learning curve.

*Additional discussion: correcting training data for trivial sequence correlations*

Preparing training/test dataset splits that are representative of practical future use cases is a substantial challenge in machine learning. While assembling test datasets from random examples of the complete dataset is the simplest approach, random train/test splitting is rarely reflective of reality. Novel datasets on which one wishes to apply a pretrained machine learning model often arrive months or years after training, and such data can hold variations from the base problem that do not conform to the statistical distribution of the training data. For example, in the case of HLA-peptide complex immunogenicity prediction, novel antigen peptides could be restricted to related but understudied HLA alleles, or be derived from emerging pathogens, new patient cohorts, or evolving cancer cell clones that differ in significant ways from our present training set. Machine learning models assessed on random training/test dataset splits are frequently published with great promise but often fail to meet expectations in future applications. Models trained on random splits of HLA-restricted peptides might produce seemingly excellent results but learn little more than trivial sequence correlations present in the available data. If data are time-stamped, one can avoid some of the pitfalls of random splits by leveraging “chronological“ dataset splits. Chronological splitting procedures involve training on data generated earlier in time and testing on more recent data points. Chronological splits directly simulate the application of pretrained machine learning models to future problems, with the disadvantage that experimental pathways used to generate training data may not be repeated in the future.

A more mathematically rigorous approach to split debiasing involves the explicit generation of training and test dataset probability distributions with non-trivial overlap. Monte Carlo simulations, first conceived in statistical mechanics for sampling low free energy states in molecular systems, have emerged as a general framework for sampling probability distributions with flexible constraints. In the case of dataset splitting for HLA-peptide complex immunogenicity prediction, we wish to minimize trivial classification performance derived from simple sequence similarities within our dataset. We can apply this constraint through the definition of a cost function, or energy function, *E:*

$$E=\mathrm{mean}\left( na{\text{-}seq}_{sim} \right)+w_{pos}*\max\left( {P1}_{sim},{P3}_{sim},\ldots,{P9}_{sim} \right) (1)$$

with the first term accounting for the mean similarity between sequences at non-anchor positions and the second term accounting for the maximum sequence similarity at any one peptide position. The second term is needed because even in cases in which mean sequence similarity is minimized, similarity can remain high at a single peptide position and lead to trivial classification results. The positional weight, *w_pos_*_,_ determines the relative importance of each energy term; a value of 0.5 was generally used in this work.

Monte Carlo simulations for correcting trivial sequence correlations are run with trial sequence exchanges between training and test sets, with acceptance dictated by a standard Metropolis criterion:

$$P\left( \mathrm{accept} \right)=\exp\left( -\beta\left[ E_{new}-E_{old} \right] \right) (2)$$

The exponential parameter β represents at artificial temperature that allows for sampling of diverse sequence sets rather than steepest-descent minimization of sequence similarities.

This general Monte Carlo procedure for was used on the full 2883 peptide dataset (seeded with a random 2308/575 peptide train/test split) to create the “debiased” training and test sets mentioned above. Sequence exchange trials were conducted with 1-1 sequence swaps between training and test sets. The performance of both the sequence and MD-graph models declined on this set by approximately three points in AUC on this debiased set, as compared to the mean random result.

*Comparison with external immunogenicity prediction tools*

| **Model** | **AUC** | **Score Type** |
| --- | --- | --- |
|  |  |  |
| netMHCpan4.0 | 0.19 | netMHCave |
| MHCflurry | 0.14 | mhcflurry_presentation_score |
| PRIME | 0.27 | prime_Score_A0201 |
| HLAthena | 0.24 | HLAthena_MSi_A0201 |
| mixMHCpred | 0.19 | mixpred_Score_A0201 |
|  |  |  |
| netMHCpan4.0(+NB) | 0.72 | netMHCave |

**Table S1. Immunogenicity prediction AUC (AUROC) results predicted on our labeled 2883 peptide dataset using five common external tools.** AUCs are computed using representative scoring functions output by each model. The result in the bottom row (netMHCpan4.0(+NB)) reflects the recovery of netMHCpan4.0 performance after the injection of 5661 peptides with negative MHC binding assay results in the IEDB.

*Additional supplementary figures*


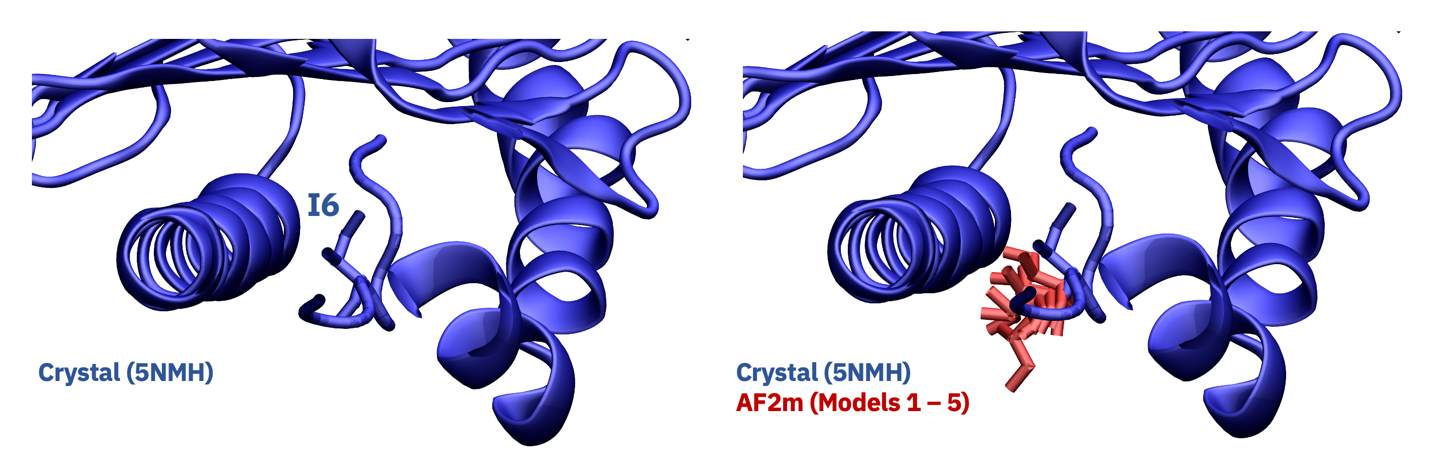


**Fig. S7. AlphaFold2-multimer (AF2m) results for prediction of 5NMH crystal template structure.** Left: crystal presentation mode for SLYNTIATL peptide in PDB structure 5NMH, including buried orientation of residue I6. Right: overlay of 5NMH structure with orientations of I6 side chain predicted by AlphaFold2-multimer. All predicted structures were assigned nearly degenerate pLDDT scores ([88-89]). Note that no significant deviations from the 5NMH structure were observed for the HLA alpha or beta-2 microglobulin domains in AF2m predictions.


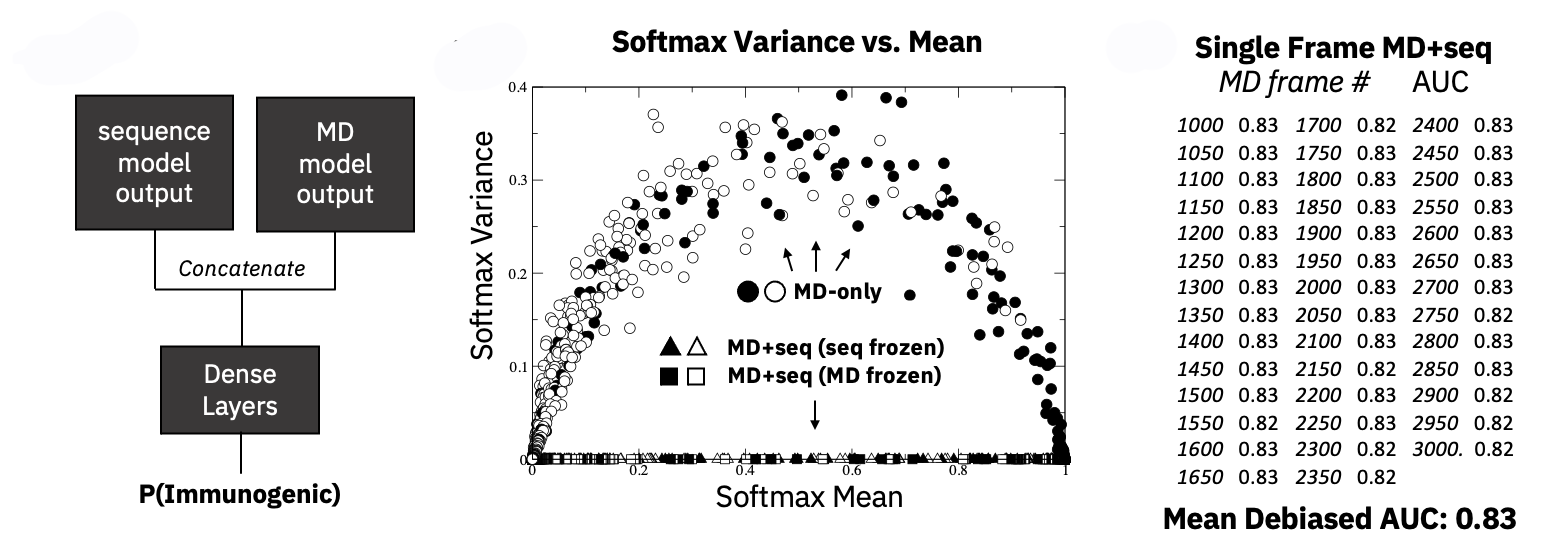


**Fig. S8. Initial attempt at multi-modal fusion (MMF) for a combined MD + sequence immunogenicity architecture.** Left: schematic of simple MMF approach involving concatenation of separate sequence model and MD-graph model representation vectors to yield a fused representation vector. That vector is fed through dense neural network layers and softmax layer to produce a joint immunogenicity prediction. Middle: Illustration of model prediction (softmax) outputs for the MD-graph model and two example models in which either the sequence or MD-graph component is trained and frozen prior to training the second component. Shaded shapes represent values for immunogenic peptides and empty shapes represent values for non-immunogenic peptides. In both fused cases, the framewise variance tied to the MD-graph model (which is largest close to the class boundary and could be useful to augment sequence features) is decimated upon of inclusion of sequence features, possibly explaining a collapse onto sequence-only model results. Right: Current best attempt at circumventing collapse of MD + sequence model predictions onto sequence model predictions. Individual models are trained on frozen sequence features and MD frames from specific timepoints; results from these framewise models are then averaged to produce a mean AUC of 0.83 on the (full) debiased test set. Since this value is not significantly better than the MD-graph result of 0.81 at 95% confidence, we do not argue this current attempt provides a significant improvement over the other approaches.

*Discussion of future directions*

We have limited our study to peptides restricted to the class-I HLA-A02 supertype (one of the most data-rich and well-studied supertypes) and peptides drawn from pathogen and human host sources. Extension of MD-graph immunogenicity prediction models to the other dozen or so class I HLA-A and HLA-B supertypes can likely be achieved through transfer of graph representation weights derived from this study. However, distinct properties of anchor binding pockets and peptide anchor residues in other supertypes could significantly impact dynamics at the peptide termini. Furthermore, structural variations across HLA alleles that affect the morphology and accessibility of the peptide binding groove could impact the nature of peptide presentation modes that give rise to immunogenicity. Accordingly, the extent of transferability of graph representations for general immunogenicity prediction remains an unknown quantity. Application of MD-guided immunogenicity prediction to class II HLA is also a topic of interest, though the additional complications of longer and more flexible peptides with more diverse HLA binding registers increase the complexity of the problem.

The present work uses immunogenic peptides to pathogen sources. Our results here raise the possibility of applying similar approaches to cancer neoantigen and autoimmune antigen peptide systems. A preponderance of cancer neoantigens that bind HLA-I molecules also tend to be non-immunogenic, and differentiation of immunogenic neoantigen binders from non-immunogenic binder counterexamples has proven a difficult problem. Though recent strides toward success should be acknowledged, it is perhaps unsurprising that neoantigen immunogenicity prediction has proven challenging, considering cancer neoantigens are generally only small perturbations on host peptides that are conditioned to be non-immunogenic. Moreover, neoantigens that are strongly immunogenic are sometimes derived from host peptides that are themselves aberrantly immunogenic, maintaining the subtle distinction between classes. Our MD-graph model was able to perform relatively well on small pathogen datasets that are difficult for our sequence benchmark to classify. Whether such relative performance can be transferred to neoantigen sets of similar size is an open question and worthy of future investigation.

An additional advantage of applying MD-based architectures to immunogenicity prediction concerns the simulation output: since MD trajectories are required to generate features for our MD-graph model, later immunogenicity predictions are accompanied by detailed structural and dynamical representations of peptide conformations presented for T cell recognition. If one wishes to design therapeutics on the other side of the immune synapse – for example, therapeutic TCRs delivered through engineered T cells or antibodies/antibody fragments designed with analogous therapeutic intent – one can use the output of MD-based immunogenicity predictions as a target structure starting point. Classifiers analogous to immunogenicity prediction models can be trained on three-body HLA-peptide-TCR or HLA-peptide-antibody complexes, with the goal of optimizing binding affinity; training can be driven by labels derived from *in-silico* free energy calculations, if desired.

MD data can be used to train AI models that produce similar types of simulation data. More advanced generative dynamics models (e.g., Markov models, in a future form) could potentially be trained to serve as MD simulator surrogates, outputting molecular data that could feed immunogenicity and affinity classifiers at greatly accelerated pace and significantly lower cost.

Foundation model approaches for protein sequence representation are primed to improve method transferability across a range of biological systems. AI methods that leverage physics-based simulations, therefore, cannot be developed in a vacuum separate from the sequence modeling field, and models that combine the most useful aspects of MD-graph and sequence immunogenicity prediction are paramount targets for future research. While we limited our present study to a single sequence model benchmark, multimodal MD+seq models should also be explored in the full context of the plethora of available sequence classifiers for immunogenicity prediction. General approaches to multimodal fusion, which concerns the productive combination of diverse feature types within deep learning models, may also need to improve to reach these goals.
